# CDCP1/mitochondrial Src axis increases electron transport chain function to promote metastasis in triple-negative breast cancer

**DOI:** 10.1038/s41416-025-03163-6

**Published:** 2025-09-04

**Authors:** Jordan A. Woytash, Austin E. Y. T. Lefebvre, Ziang Zhang, Binzhi Xu, Stephanie A. Harchenko, Hoa T. Le, Andrew R. McColloch, Xiaoyu Shi, Michelle A. Digman, Olga V. Razorenova

**Affiliations:** 1 Department of Molecular Biology and Biochemistry, University of California, Irvine, CA USA; 2 Department of Biomedical Engineering, University of California, Irvine, CA USA; 3 Department of Developmental and Cell Biology, University of California, Irvine, CA USA; 4 Department of Chemistry, University of California, Irvine, CA USA; 5 Department of Biomedical Chemistry, University of California, Irvine, CA USA

**Keywords:** Breast cancer, Cancer metabolism

## Abstract

**Background:**

Triple-negative type of breast cancer (TNBC) has limited therapeutic options and frequently metastasizes, leading to low survival rates. Oxidative phosphorylation (OXPHOS) is a driver of TNBC metastasis, but the signaling underlying this metabolic change is poorly understood.

**Methods:**

We performed metabolic assays and assessed migratory and metastatic potential in cells with manipulated CDCP1/mitochondrial Src signaling.

**Results:**

We show that the pro-metastatic cell surface protein CUB-domain containing protein 1 (CDCP1) activates Src kinase localized in mitochondria, which potently induces OXPHOS and TNBC migration. Genetic targeting of either CDCP1 or mitochondrial Src, as well as pharmacological inhibition of Src reduce OXPHOS in vitro. We further show that mitochondrial Src increases OXPHOS by stimulating Complex I activity in the electron transport chain. Importantly, rescuing Complex I activity in cells devoid of CDCP1/mitochondrial Src signaling restores both OXPHOS and migration. We also provide evidence that NAD^+^ pool generated by Complex I is contributing to the observed migratory phenotype. Lastly, we determined that inhibiting mitochondrial Src reduces metastasis in TNBC cells.

**Conclusions:**

Both CDCP1 and mitochondrial Src represent potential therapeutic targets to inhibit OXPHOS-mediated TNBC metastasis.

## Introduction

Currently, breast cancer is the most diagnosed form of cancer amongst American women [1]. Most subtypes of breast cancer are driven by estrogen receptor alpha (ERα), progesterone receptor (PR), and/or human epidermal growth factor receptor (HER2) cell-surface receptors, which are targeted by standard of care therapeutic agents, inhibiting tumor growth and metastasis, and prolonging patient survival [2]. Triple negative breast cancer (TNBC) does not express any of these three receptors, thus leaving TNBC patients without targeted therapeutics [2]. TNBC is also more aggressive and metastasizes more rapidly than other breast cancer subtypes [2]. Accordingly, the 5-year survival rate drops from 91.2% for localized TNBC to 11.5% for metastatic disease [3]. Moreover, once TNBC develops resistance to chemotherapy, there are no other therapeutic options available. Thus, development of novel therapeutic strategies is of primary importance.

While increased glycolysis (Warburg effect) is an accepted hallmark of cancer, recent evidence shows that oxidative phosphorylation (OXPHOS) and fatty acid oxidation (FAO) are necessary for progression and metastasis of multiple cancers [4], including TNBC [5–9]. Key in vivo studies have demonstrated that mitochondrial biogenesis, increased OXPHOS and ATP synthesis are crucial for TNBC metastasis [5, 7, 10]. We have previously reported that CUB-domain containing protein 1 (CDCP1) is overactive in TNBC compared to normal tissue [11] and promotes metastasis in TNBC by stimulating FAO and OXPHOS, though the mechanism remains incomplete [12].

CDCP1 is located at the cell surface and serves as an adaptor protein facilitating phosphorylation of the proto-oncogene Src kinase and Src’s substrates [11, 13, 14]. CDCP1 acts as a docking site for Src and Src substrates. Once CDCP1 becomes cleaved, shedding a part of its extracellular domain, it dimerizes, binds to Src, and brings two Src molecules into close proximity to each other, causing transphosphorylation and Src activation. Similar mechanism operates with Src substrates (such as EGFR and HGF), where Src and its substrates bind to CDCP1 dimer and are brought into close proximity, causing substrate phosphorylation by Src [11, 14–16]. CDCP1 and/or Src signaling has been reported to potentiate metastasis in multiple mouse models of cancer including breast, prostate, melanoma and colorectal cancers [12, 14, 17–21]. Src kinase is a known OXPHOS regulator [22–34]. Despite lacking a canonical mitochondrial targeting sequence (MTS), Src has been reported to localize in mitochondria in several cellular contexts [27, 31, 32, 35–37], where it has been linked to OXPHOS regulation [27–37]. Specifically, mitochondrial-localized Src is reported to phosphorylate tyrosine residues of the electron transport chain (ETC) subunits to stimulate ETC function and OXPHOS [28, 30–34]. Based on the above, here we investigated the CDCP1 signaling towards mitochondrial-localized Src and their connection to OXPHOS and TNBC cell migration.

Our data provide a molecular mechanism of elevated OXPHOS in TNBC via CDCP1/mitochondrial Src pathway. Importantly, our data specifically show that this pathway activates Complex I. Moreover, we show that NAD^+^ produced by Complex I is important for TNBC migration. Thus, our data position CDCP1/mitochondrial Src pathway as therapeutic targets to reduce Complex I activity and OXPHOS in TNBC. Since OXPHOS/metastasis pathway has been reported in other types of cancer besides TNBC, including pancreatic, ovarian, melanoma and prostate cancers [38–42], the CDCP1/mitochondrial Src pathway needs to be further investigated in these cancer cell contexts.

## Materials and methods

### Cell lines, media, treatment with Src inhibitors

MDA-MB-231, SUM159, HEK293T, MDA-MB-468 and UCI-082014 [11] cell lines were grown in Dulbecco’s Modified Eagle Medium (DMEM) (Genesee Scientific #25-500) with 10% Fetal Bovine Serum (FBS) (Genesee Scientific #25-514), 100 u/mL penicillin and 100 μg/mL streptomycin (1% P/S). Cells were grown in mixed gas CO_2_ water-jacketed incubators (21% O_2_, 5% CO_2_) and regularly confirmed to be negative for mycoplasma contamination. TNBC cells were treated with 1 μM Saracatinib (Cayman Chemical Company, #11497), 10 μM Dasatinib (Enzo Life Sciences, #76003-104), 0.5 mM nicotinamide riboside (Cayman Chemical Company, #23132) or 0.5 mM nicotinamide mononucleotide (Cayman Chemical Company, #16411) for 16 hours unless noted otherwise.

### Lentivirus production and infection of target cells

Detailed protocol is described in Razorenova et al. [43]. Lenti-X 293T cells were transfected with lentiviral plasmids for shRNA [11] or gene expression and packaging plasmids, pVSVG and ΔR8.2. TNBC cells were treated with virus-containing media supplemented with 6 µg/mL of polybrene, followed by selection in 1 µg/mL puromycin- or 2 µg/mL blasticidin-containing media for a minimum of 1 week. MTS-Kd-Src-Flag-Puro lentiviral expression vectors were packaged into lentiviral particles purchased from VectorBuilder Inc, Chicago, IL USA (VB240904-1416wze).

### Generation of CDCP1 deficient cell lines

LentiGuide-puro and LentiCas9-blast viruses were produced in Lenti-X cells as described above. Three different LentiGuide-puro viruses were produced to target CDCP1 (constructs were generated by cloning using the following self-annealing oligo pairs: gCDCP1#1 5’caccggggtctctatcgcactgcta + 5’aaactagcagtgcgatagagaccc; gCDCP1#2 5’caccgcgatagagaccccgcagttc + 5’aaacgaactgcggggtctctatcg; gCDCP1#3 5’caccggtaggcaacaacgatgtcga + 5’aaactcgacatcgttgttgcctac, sequences complementary to CDCP1 gene are underlined). The LentiGuide-puro virus was produced to target GFP (and served as a non-targeting control), the sequence complementary to GFP is as follows: 5’ ggcgaggagctgttcaccg. First, cells were transduced with LentiCas9-blast and selected with blasticidin at 2 µg/mL. Second, cells were transduced with LentiGuides and selected with puromycin at 1 µg/mL. Third, CDCP1 knockout cells were enriched by fluorescence-activated cell sorting (FACS) of cells with no CDCP1 expressed on the cell surface. Antibodies are listed in Supplemental Table 1. Stable CDCP1 knockdown (shCDCP1) and control (shGFP) cell lines were created as previously described [11], and pLKO.1shGFP was a kind gift from Silvestre Vicent (Stanford University).

### Western blot analysis

The western blot protocol was adapted from Razorenova et al. [44]. Cells were lysed in lysis buffer (20 mM Tris-HCl [pH 7.5], 150 mM NaCl, 1 mM EDTA, 1 mM EGTA, 1% Triton X100, 2.5 mM Na_4_P_2_O_7_, 1 mM β-glycerophosphate, 1 mM Na_3_VO_4_) with protease inhibitors (Fisher Scientific, Pittsburgh, PA #P1-88266) and phosphatase inhibitors (Roche #04906845001). Fifty micrograms of protein were used per lane. Antibodies are listed in Supplemental Table 1.

### Seahorse XF24 respirometry

The Oxygen Consumption Rate (OCR) was measured using a Mito Stress Test Kit and XF24 Extracellular Flux Analyzer (Agilent) according to the manufacturer’s protocol. In brief, 80,000 MDA-MB-231/UCI-082014 cells or 50,000 SUM159/HEK293T cells were plated in 100 μL of their standard growth media and cultured overnight. HEK293T cells were plated on poly-L-lysine (0.1 mg/ml) to improve attachment. Next day, cells were washed with XF base medium supplemented with 17.5 mM glucose, 2 mM L-Glutamine and 10 mM sodium pyruvate and incubated in a CO_2_-free incubator at 37 °C for 1 hour to equilibrate prior to loading. OCR measurements were taken before and after the addition of oligomycin (ATP synthase inhibitor, 1 μM), FCCP (mitochondrial oxidative phosphorylation uncoupler, MDA-MB-231/UCI-082014 = 1 μM, SUM159 = 0.5 μM, HEK293T = 0.25 μM), Rotenone/Antimycin A (Complex I and III inhibitors, respectively, 0.5 μM each), and used to calculate basal respiration, maximal respiration and ATP production. OCR rates were normalized to cell numbers. For Complex I activity, cells were prepared as described above, and OCR was measured before and after the addition of 0.5 μM rotenone [45–48]. Complex I activity was determined by subtracting the lowest OCR measurement post-rotenone from the last basal OCR measurement pre-rotenone and normalized to cell number.

### NADH Fluorescent Lifetime Imaging (FLIM)

NADH fluorescence lifetime is used to determine its enzyme-bound (active) or free (inactive) state. A higher ratio of bound/free NADH corresponds to higher OXPHOS. The NADH FLIM imaging experiments were carried out as previously described [10]. In short, the FLIM images were obtained using an inverted laser scanning confocal microscope (LSM 710) with a ×40/1.2 numerical aperture C-Apochromat water-immersion objective, with the cells maintained in a stage-top incubator at 37 °C and 5% CO_2_ throughout imaging. NADH in MDA-MB-231 cells was excited using a two-photon Ti:Sapphire laser (Spectra-Physics, MaiTai) with an 80 MHz repetition rate at 740 nm and at ~2 mW power at the sample. This signal was passed through a 690 nm dichroic filter to separate excitation and emission signals. The fluorescence emission was separated by a bandpass filter (442/46 nm) to capture the cell auto-fluorescence and exclude any mApple emission and was then detected using a photomultiplier tube (Hamamatsu, #H7422P-40). The fluorescence lifetime decays were captured in the frequency domain using an A320 FastFLIM box (ISS) which were then mapped onto the phasor plot using the SimFCS 4 software—developed at the Laboratory for Fluorescence Dynamics at the University of California, Irvine—for quantitative NADH lifetime analysis. The lifetime free and protein bound form of NADH were used to determine the metabolic trajectory (the “M-Trajectory”) on phasor FLIM plot, as demonstrated by Stringari et al. [49]. The instrument response time was calibrated before each experiment by imaging Coumarin-6 in ethanol (~10 μM), which has a known single exponential fluorescence lifetime of 2.4 ns.

### NAD^+^/NADH assay

The NAD^+^/NADH ratio was determined following the manufacturer’s protocol (Millipore Sigma MAK460).

### ATP luminescent assay

The Luminescent ATP Detection Assay was performed following the manufacturer’s protocol (Abcam #ab113849). Luminescence read with Bio Tek Cytation5 microplate reader (Agilent) was normalized to cell number.

### Immunofluorescent staining

Cells were fixed with 4% paraformaldehyde for 10 min, then treated with 0.25% Triton X-100 in 1xPBS for 5 min and blocked for 1 h in 3% bovine serum albumin (BSA) in 1xPBS. Slides were then incubated overnight in primary antibody at 4 °C, followed by incubation for 2 h in secondary antibody at room temperature in the dark. Antibodies are listed in Supplementary Table 1. Both primary and secondary antibodies were diluted in 3% BSA in 1xPBS. Coverslips were mounted with 1xPBS. Cells were imaged using a ZEISS Elyra 7 super-resolution microscope with Lattice SIM². Colocalization analysis and quantitation were performed using the surface-to-surface algorithm in Imaris Microscopy Image Analysis Software. The total Src volume and total mitochondrial volume were masked as “surfaces.” The shortest distance between surfaces was quantified, and only surfaces that were within 0.3 microns were chosen (as tomm20 is an outer mitochondrial matrix protein and MTS-Src-Flag constructs are localized to the mitochondrial matrix, we don’t expect a perfect overlap. The same logic applies to endogenous Src localization. The diameter of mitochondria is anywhere from 0.5 to 1.5 micron [50, 51], so 0.3 micron is a conservative constraint). The total volume of colocalizing surfaces as well as the total Src volume was quantified and the percentage of total Src in the mitochondria was then calculated using 5–8 fields of view. To assess CDCP1 localization, cells were prepared as described above and the Zeiss LSM 780 confocal microscope was used to image CDCP1 and mitochondria (using Tomm20 as a mitochondrial marker).

### Isolation of crude mitochondrial fractions

To prepare mitochondrial and cytosolic fractions, cells were harvested, washed in ice-cold PBS, minced and then resuspended in homogenizing buffer (20 mM HEPES-KOH, pH 7.5, 10 mM KCl, 1.5 mM MgCl_2_, 1 mM sodium EDTA, 1 mM sodium EGTA, and 1 mM dithiothreitol) containing 250 mM sucrose and a mixture of protease inhibitors (1 mM PMSF, 1% aprotinin, 1 mM leupeptin, 1 mg/ml pepstatinA, and 1 mg/ml chymostatin). After 30 min incubation on ice, tissues were homogenized using a glass Pyrex homogenizer (type B pestle, 50–60 strokes) and centrifuged at 1000 × *g* for 5 min at 4 °C to remove debris and unbroken cells. The resulting supernatant was centrifuged at 12,000 × *g* for 12 min to obtain mitochondria-enriched preparation as a pellet. The supernatant is the cytosolic fraction. The mitochondrial pellet was washed 3 times in homogenizing buffer. 5–10 μg of total protein, cytosolic and mitochondrial fractions were analyzed by western blot as described above.

### Co-immunoprecipitation

Cells were lysed as described in western blotting. 15 μL of Flag magnetic beads (Millipore Sigma #M8823) were added to 250 μg of protein in 250 μL of cell lysis buffer and incubated at room temperature with gentle agitation for 3 h. Immunoprecipitated proteins were collected using a magnetic stand, washed 5 times for 5 min with cell lysis buffer without protease or phosphatase inhibitors, boiled in 2× sample loading buffer (125 mM Tris-HCl pH 6.8, 4% SDS, 0.02% bromophenol blue, 20% glycerol, 4% mercaptoethanol) for 6 min and analyzed by western blot.

### Cell transient transfections with siRNAs and cDNAs

MDA-MB-231 cells were transfected using Lipofectamine 3000 reagent (Thermo Fisher Scientific #L3000008) following the manufacturer’s protocol. SUM159 and HEK293T cells were transfected using Lipofectamine and Plus reagents (Thermo Fisher Scientific #18324012 and #11514015) following the manufacturer’s protocol. ON-TARGETplus smartpool of 4 siRNAs targeting Src and CDCP1 as well as a non-targeting pool (#L-003175-00-0005, #L-010732-00-0005, and #D-001810-10-05) were purchased through Horizon Discovery. Mitochondrially-localized FLAG-tagged WT and kinase dead Src (pcDNA3-MTS-WT-c-Src-FLAG and pcDNA3-MTS-KD-c-Src-FLAG, respectively), and constitutively active Src (pEF6-Src-Y530F) were purchased from Addgene (# 44652, # 44653, and #124659) [33, 52]. Fl-CDCP1 and cCDCP1 (pLM-CMV-flCDCP1-puro and pLM-CMV-cCDCP1-puro, respectively) were described in Wright et al. [11]. pWP1-EV-GFP and pWPI-NDI1-GFP were provided by Dr. Chandel [53].

### Mitochondrial ROS staining

Mitochondrial-specific ROS was detected using MitoSOX Red reagent and was performed following the manufacturer’s protocol (Thermo Fisher, #M36008). Mitochondrial ROS was quantified in individual cells on the BD Fortessa X20.

### Cell migration

Cells were treated with 100 μM mitomycin C for 2.5 h before being detached with 0.05% trypsin in 1×PBS. Thirty thousand SUM159 cells in serum-free DMEM were added to the top chamber of the transwell inserts (Corning, NY, USA; #3464) and immersed in 10% FBS-containing media in the bottom chamber. Cells were allowed to migrate for 24 h. Cells from the top of the transwell inserts were removed with a cotton swab and cells that migrated through the transwell inserts were fixed, and fluorescent microscopy was used to count the GFP-positive cells. Cells were counted in 6–7 fields of view per transwell at 20× and normalized to control to quantify average fold change (FC) in migrated cells.

### 3D invasion assay

Cells were plated at 5000 cells per well in 1:1 mix of matrigel (growth factor reduced) (Corning, Cat. No. CB40230) and EpiCult^TM^ -B Media supplemented with 5% FBS, 1% P/S, 10 ng/mL human epidermal growth factor, and 10 ng/mL basic fibroblast growth factor. Cells were allowed to grow and form spheres for 7 days.

### MDA-MB-231 in vivo assays

1.0 ×10^6^ gGFP, gCDCP1-1, gCDCP1-3, EV-puro or MTS-Kd-Src-flag-puro cells were injected into the 4^th^ mammary fat pad of 8-week-old female NSG mice (*N* = 6, 5, 6, 7, 7 per group, respectively). Mouse sample size for metastasis experiments was consistent with our previous published work [12]. No randomization was used. For gGFP and gCDCP1 cells, cells were injected bilaterally to produce two tumors per mouse and combined tumor volume was monitored. Caliper measurements of tumors were taken twice a week and tumor volume was calculated as Volume = Length × Width^2^ × 0.51. Tumors were resected when volume reached 1.0 cm^3^ (30 days post-injection). Analysis of metastasis in lung tissue was performed as previously described [54]. Briefly, the lungs of NSG mice were harvested, dissociated into single cells, and stained with fluorescently conjugated antibodies for CD298 (Biolegend, Cat. no. 341704) and MHC-I (eBioscience, Cat. no. 17-5957-82), and flow cytometry was used to analyze and quantify disseminated CD298^+^/MHC-I^−^ MDA-MB-231 cells using the BD Fortessa flow cytometer. All mouse experiments were approved by the Institutional Animal Care and Use Committee at UC Irvine (#AUP-22-012).

### Statistical analysis

Statistical analysis was conducted in GraphPad Prism version 10.0 using a Student’s *t* test or ANOVA between groups with *p  <  0.05, **p  <  0.01, ***p < 0.001 and ****p < 0.0001 unless otherwise noted. Error bars (StDev or SEM) and the number of biological replicates are indicated in each figure legend.

## Results

### Cell surface protein CDCP1 activates Src localized in mitochondria

**Fig. 1:**
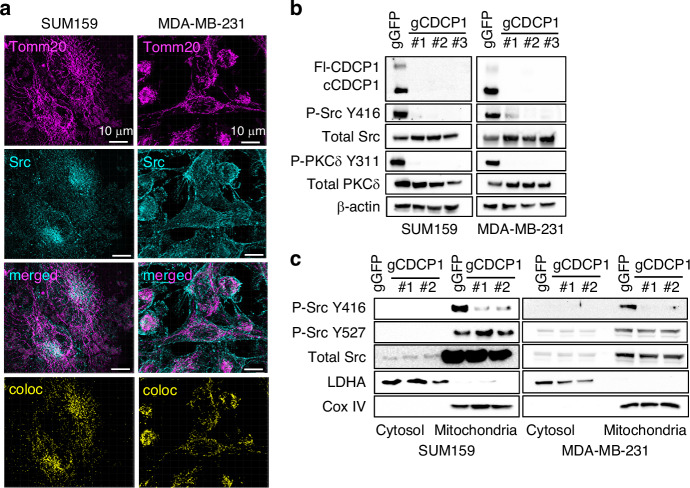
Cell surface protein CDCP1 activates Src localized in mitochondria. **a** Src is localized in mitochondria as assessed by super-resolution microscopy. Total Src is shown in cyan, Tomm20 (mitochondrial marker) is shown in magenta. Mitochondrial-localized Src (Tomm20 and Src colocalization [coloc]) was quantitated by surface-to-surface analysis, averaging 4–6 fields of view and is shown in yellow. Scale bar = 10 microns. **b** Western blot showing that CDCP1 knockouts (gCDCP1 #1-3) abrogate Src activity as judged by its autophosphorylation at Y416 and phosphorylation of Src’s substrate–PKCδ–at Y311. Fl- and cCDCP1 refer to full length and cleaved CDCP1. **c** Western blot showing that active Src phosphorylated at Y416 is detected in the mitochondrial fraction, and its phosphorylation at Y416 is abrogated by CDCP1 knockouts. Subcellular fractionation was confirmed by a cytosolic-specific marker LDHA and a mitochondrial-specific marker COXIV. *n* = 2. In (**b**, **c**) two to three different gRNAs were used to knockout CDCP1 with CRISPR/Cas9; gRNA targeting GFP was used as a control. All experiments were conducted in two TNBC cell lines as indicated.

Since CDCP1 is a known upstream regulator of Src kinase [11, 13, 14], and mitochondrial-localized Src regulates OXPHOS [27–37], we hypothesized that a portion of active Src protein is targeted to mitochondria in TNBC. We further hypothesized that Src activity in mitochondria is regulated by CDCP1. We obtained super-resolution 3D images and quantitated the percentage of the total volume of endogenous Src localized to the mitochondria: 21.1 ± 6.9% in MDA-MB-231 and 20.6 ± 4.4% in SUM159 TNBC cells (Fig. 1a). In parallel, we have shown that Src activator CDCP1 [14] does not localize to mitochondria but localizes at the cell surface and in endosomal vesicles (Supplementary Fig. 1A, B). To assess the impact of CDCP1 loss on Src activity in mitochondria, we developed several CDCP1 knockout TNBC cell lines using CRISPR/Cas9. We confirmed the loss of CDCP1 expression accompanied by the loss of its downstream signaling axis—phosphorylation of Src and PKCδ (Fig. 1b). Interestingly, even though CDCP1 does not localize to the mitochondria, CDCP1 knockout almost completely abrogated mitochondrial Src phosphorylation at Y416 (CDCP1-dependent activation mark [14, 15]), while Src phosphorylation at Y527 (CDCP1-independent phosphorylation mark) remained unaltered (shown by subcellular fractionation, Fig. 1c). Our cell fractionation procedure did not include a membrane fraction, where CDCP1-dependent Src phosphorylation at Y416 has been primarily reported [55–59], and the cytoplasmic fraction, as expected, lacks active Src. Together these data show that Src activity in mitochondria of TNBC cells is regulated by CDCP1.

### Both CDCP1 and Src increase OXPHOS in TNBC

**Fig. 2:**
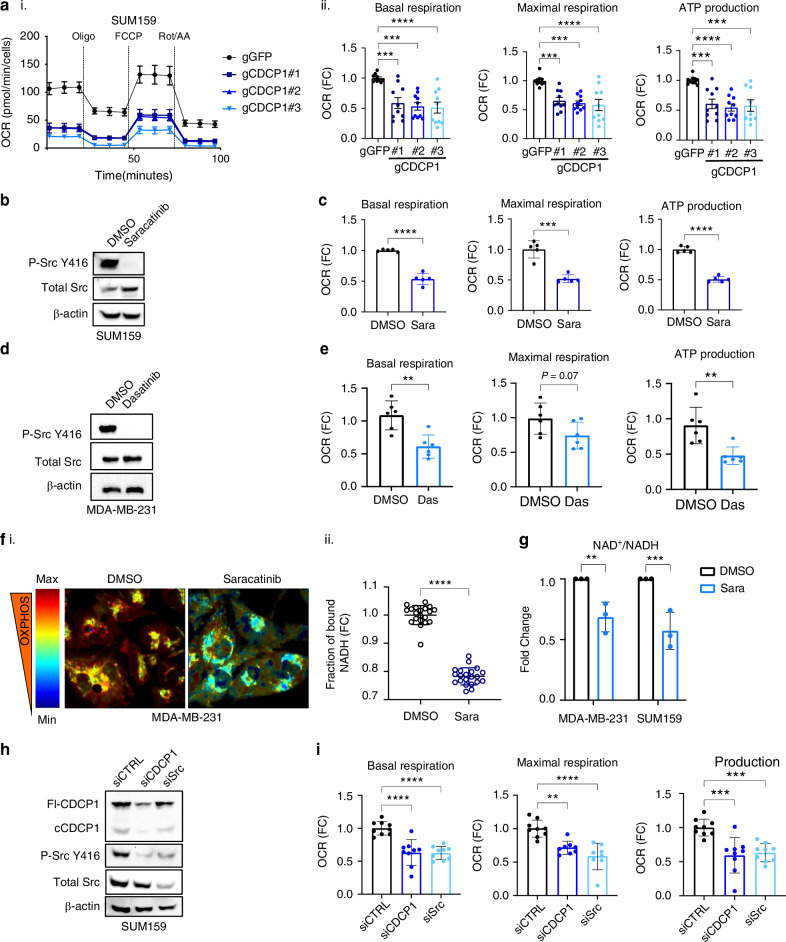
Both CDCP1 and Src increase OXPHOS in TNBC. **a** CDCP1 knockout reduces OXPHOS in SUM159 cells as measured by Seahorse XF24 analyzer. Oxygen consumption rate (OCR) is shown in response to treatments with Oligomycin (Oligo), carbonyl cyanide-p-trifluoromethoxyphenylhydrazone (FCCP), and Rotenone/Antimycin A (Rot/AA). Basal and maximal respiration were determined by Seahorse Mito Stress Test analysis in addition to ATP production. i) Representative OCR curve and ii) quantitation (*n* = 10 per condition). **b** Western blot showing Src inhibition by treatment with 1 µM Saracatinib for 16h–phospho-Src Y416 is reduced. **c** Src inhibition by Saracatinib (Sara) treatment reduces OXPHOS in SUM159 cells (*n* = 5 per condition). **d** Src inhibition in MDA-MB-231 cells by treatment with 10 µM Dasatinib for 16h–phospho-Src Y416 is reduced. **e** Src inhibition by Dasatinib (Das) reduces OXPHOS in MDA-MB-231 (*n* = 6 per condition). **f** Src inhibition by Saracatinib reduces OXPHOS in MDA-MB-231 as measured by NADH FLIM. i) Representative NADH FLIM images and ii) quantitation. All images were taken using the 2× zoom on the 40× objective. Each dot represents a single cell, error bars represent SEM. **g** Src inhibition by Saracatinib reduces NAD^+^/NADH ratio. **h** Western blot showing siRNA-mediated Src and CDCP1 knockdowns, accompanied by a reduction in phospho-Src Y416. **i** Both CDCP1 and Src knockdowns reduce OXPHOS in SUM159 (*n* = 8–9 per condition). Seahorse experiments in (**c**, **e**, **i**) were conducted as described in (**a**) (FC fold change). *P* values in (**a**ii, i) were analyzed by one way ANOVA with multiple comparison post hoc analysis, error bars represent SEM (**a**) and StDev (**i**). *P* values in (**c**, **e**, **f**ii, **g**) were analyzed by Student’s *t* test and error bars represent StDev (**c**, **e, g**) and SEM (**f**ii). **P < 0.01, ***P < 0.001, ****P < 0.0001. FC fold change.

We used CDCP1 knockout cells generated in Fig. 1b to assess the role of CDCP1 in OXPHOS. CDCP1 knockout cells (gCDCP1) have reduced basal and maximal respiration, as well as ATP production in three TNBC cell lines as compared to gGFP control as determined by the Seahorse Mito Stress Test analysis (Fig. 2a and Supplementary Fig. 2A–C). Concomitant with these results, we also observed a decrease in the NAD^+^/NADH ratio in CDCP1 knockout cells (Supplementary Fig. 2D). Since Src has been reported to regulate OXPHOS [6, 23, 25, 26, 31–34], and we have shown that CDCP1 activates Src in mitochondria, next we assessed the impact of inhibiting Src activity pharmacologically and genetically on OXPHOS in TNBC. Treatments with a specific Src family kinase inhibitor—saracatinib—or a broad tyrosine kinase inhibitor—dasatinib—significantly reduced OXPHOS in TNBC cell lines as assessed by the Seahorse Mito Stress Test analysis (Fig. 2b–e, Supplementary Fig. 3A–E). NADH fluorescence lifetime imaging (NADH FLIM) analysis relying on ratios of enzymatically bound to enzymatically free NADH for OXPHOS measurements [60] confirmed that saracatinib treatment reduced OXPHOS in MDA-MB-231 cells (Fig. 2f). Accordingly, also observed a decrease in the NAD^+^/NADH ratio in cells treated with saracatinib (Fig. 2g). Since both dasatinib and saracatinib have off-target effects, we complemented these experiments with a genetic approach, where we transiently knocked down c-Src and CDCP1 (positive control) using siRNA smartPOOLs in SUM159 cell line (Fig. 2h). Seahorse analysis revealed that either Src or CDCP1 knockdowns significantly reduced OXPHOS (Fig. 2i, Supplementary Fig. 3F). Together, these data show that both CDCP1 and Src signaling promote OXPHOS in TNBC.

### CDCP1 increases OXPHOS via mitochondrial Src signaling in TNBC cells

**Fig. 3:**
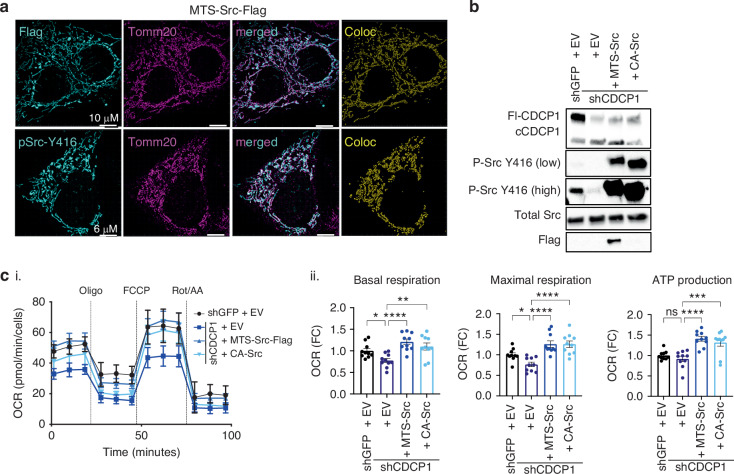
CDCP1 increases OXPHOS via mitochondrial Src signaling in TNBC cells. **a** Flag-tagged mitochondrially-targeted wild-type Src (MTS-Src-Flag) efficiently localizes to mitochondria in SUM159 cells as assessed by super-resolution microscopy. Immunofluorescence and co-localization were conducted as in Fig. 1a. Scale bars as indicated. **b** Western blot showing CDCP1 expression and Src activity (P-Y416) in SUM159 cells stably expressing shGFP or shCDCP1#1 and transiently transfected with either an empty vector-control (EV) or MTS-Src-Flag or constitutively active Src (CA-Src) expression constructs. **c** MTS-Src-Flag rescues OXPHOS in SUM159 cells where CDCP1 was knocked down. OXPHOS measured as in Fig. 2a. i) Representative OCR curve and ii) quantitation (*n* = 10 per condition). Experiments were performed at 48 h post-transfection. *P* values were analyzed by one way ANOVA with multiple comparison post hoc analysis, error bars represent SEM. *P < 0.05, **P < 0.01, ***P < 0.001, ****P < 0.0001.

Since CDCP1 activates mitochondrial Src, and both CDCP1 and Src promote OXPHOS, we hypothesized that CDCP1 regulates OXPHOS by activating mitochondrial Src. To test this, we overexpressed a wild-type Src targeted to mitochondria by mitochondria targeting sequence (MTS) attached to its N-terminus and with a Flag tag attached to its C-terminus (MTS-Src-Flag) [33], as well as a constitutively active Y530F Src (CA-Src) [52] with no restrictions on localization in SUM159 CDCP1 knockdown cells. Similar to CDCP1 knockout cells, stable CDCP1 knockdown cells significantly reduce OXPHOS (Supplementary Fig. 4A, B). Our co-localization analysis shows that the MTS-Src-Flag protein localizes to mitochondria (98.69 ± 0.87% of Flag co-localized with Tomm20) and is active (phosphorylated at Y416, Fig. 3a). While activity of endogenous Src strictly depends on CDCP1 expression [13, 14, 56, 61] (see also Figs. 1b, c, 2h), Src overexpression leads to its overactivation [62] bypassing the requirement for CDCP1, as evidenced by western blot analysis (Fig. 3b). Notably, MTS-Src-Flag rescued OXPHOS in CDCP1 knockdown cells to a similar extent as CA-Src (Fig. 3c). These data establish CDCP1/mitochondrial-Src pathway as the driver of OXPHOS in TNBC.

### Inhibition of mitochondrial Src activity reduces ATP production and OXPHOS

**Fig. 4:**
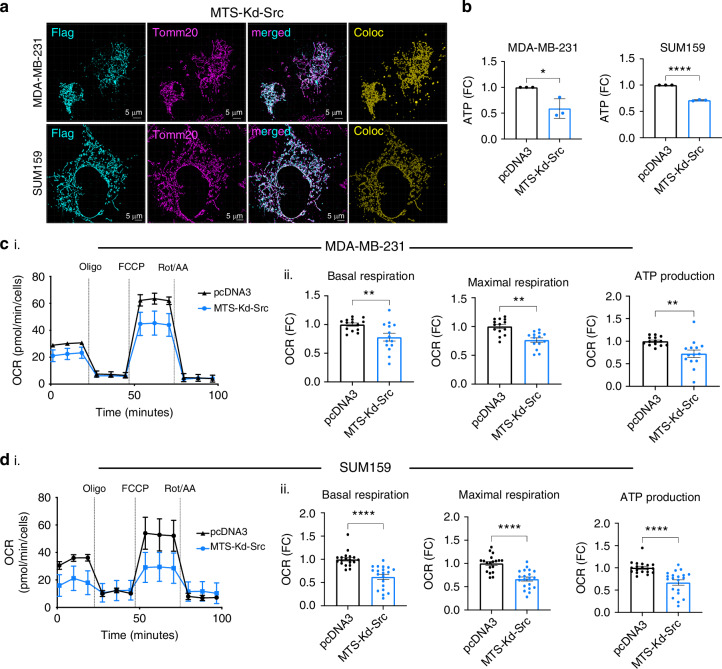
Inhibition of mitochondrial Src activity reduces OXPHOS and ATP production. **a** MTS-Kd-Src-Flag efficiently localizes to mitochondria as assessed by super-resolution microscopy. Immunofluorescence was conducted as in Fig. 1a. Scale bar = 10 microns. **b** Overexpression of MTS-Kd-Src-Flag decreases ATP levels in MDA-MB-231 and SUM159 cells as assessed by ATP luminescent assay; *n* = 3. **c**, **d** Overexpression of MTS-Kd-Src-Flag reduces OXPHOS in MDA-MB-231 and SUM159 cells. i) Representative OCR curves and ii) quantitation (*n* = 15–20 per condition). Seahorse experiments were conducted as described in Fig. 2a. All experiments in this Fig. were performed 48 h post-transfection. *P* values in (**c**, **d**ii) were analyzed via Student’s *t* test, error bars represent SEM. ****P < 0.0001

Given that CDCP1 regulates mitochondrial Src activation, we sought to determine if inhibiting Src activity specifically in mitochondria would block OXPHOS. To this end, we overexpressed a Flag-tagged kinase-dead (K298M) Src targeted to mitochondria by MTS (MTS-Kd-Src-Flag) [33] in both SUM159 and MDA-MB-231 cells. Dominant-negative MTS-Kd-Src-Flag localizes to mitochondria (98.9 ± 0.44% in SUM159 and 95.18 ± 4.69% in MDA-MB-231 of Flag co-localized with Tomm20, Fig. 4a). Inhibition of Src activity in mitochondria significantly reduced total ATP (Fig. 4b). Additionally, it reduced basal respiration, maximal respiration, and ATP production in both cell lines tested (Fig. 4c, d). Thus, blocking mitochondrial-localized Src activity reduces OXPHOS in TNBC, arguing that Src signaling in the mitochondrial compartment is responsible for high OXPHOS phenotype in TNBC cells.

### CDCP1/mitochondrial Src axis stimulates OXPHOS in TNBC via Complex I of electron transport chain

**Fig. 5:**
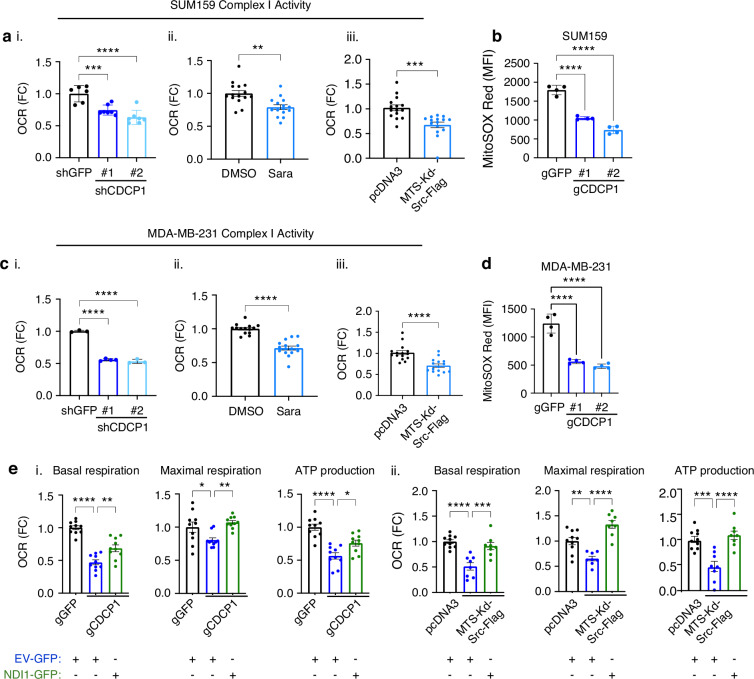
CDCP1/mitochondrial Src axis stimulates OXPHOS in TNBC via Complex I of electron transport chain. **a**, **c** CDCP1/mitochondrial Src axis inhibition decreases Complex I activity in SUM159 and MDA-MB-231 cells as determined by assessing OCR before and after rotenone (Complex I inhibitor) treatment using the Seahorse XF24 analyzer. CDCP1/mitochondrial Src axis was inhibited by two shRNAs targeting CDCP1 with *n* = 6–7 per condition (i), treatment with 1 µM Saracatinib (Sara) for 16 h with *n* = 15 per condition (ii), and overexpression of MTS-Kd-Src-Flag, measurements performed 48 h post transfection with *n* = 15 per condition (iii). **b**, **d** CDCP1 knockout reduces mitochondrial-specific ROS production as assessed via MitoSOX red staining and quantified via flow cytometry. **e** NDI1 overexpression rescues OCR in CDCP1 knockout SUM159 cells with *n* = 10 per condition (i), and in MTS-Kd-Src-Flag-transduced SUM159 cells with *n* = 8–10 per condition (ii). Measurements performed 48 h post transfection. Stable CDCP1 knockdown and knockout cell lines were described in Supplementary Fig. 4 and 1B respectively. *P* values in (**a**i, **c**i) and (**b**–**e**) were analyzed by one way ANOVA with multiple comparison post hoc T-test, while *P* values in (**A**ii-iii and **C**ii-iii) were analyzed by Student’s *t* test; error bars represent SEM; *P < 0.05, **P < 0.01, ***P < 0.001, ****P < 0.0001.

Seahorse analysis allows for specific assessment of Complex I activity, which can be calculated as the change in OCR in response to the specific Complex I inhibitor, rotenone. Knocking down CDCP1, inhibiting Src by saracatinib, as well as inhibiting mitochondrial Src activity by MTS-Kd-Src-Flag significantly reduced Complex I activity in both SUM159 and MDA-MB-231 cell lines (Fig. 5a, c, Supplementary Fig. 5). Mitochondrial Reactive Oxygen Species (ROS) are naturally generated by Complexes I and III and to a lesser extent Complex II in ETC [63, 64]. Likewise, decreased Complex I activity reduces mitochondrial ROS production [63, 65]. This is not to be confused with acute chemical inhibition of Complex I leading to destabilization of the complex, electron leak, and increase in mitochondrial ROS production [46, 66]. In line with a decreased Complex I activity in CDCP1 knockout cells, we observed a significant decrease in mitochondrial ROS in these cells as compared to gGFP controls, while overexpressing CDCP1 in HEK293T cells significantly increased the production of mitochondrial ROS (Fig. 5b, d, Supplementary Fig. 6). Mitochondrial ROS was quantified by staining with a MitoSOX Red probe that produces fluorescence upon oxidation by mitochondrial ROS [67].

Several Complex I subunits were reported to be in a complex and phosphorylated by mitochondrial Src [31, 33, 68] in other cellular contexts. Thus, we transfected MDA-MB-231 and SUM159 cell lines with a Flag-tagged wild-type Src targeted to mitochondria (MTS-Src-Flag) and assessed its interaction with several Complex I subunits: NDUFS1 [34], NDUFA8 [28], and NDUFB10 [31]. MTS-Src-Flag localized to mitochondria and was active in that compartment (p-Src Y416) as determined by super-resolution microscopy (Fig. 3a, Supplementary Fig. 7; 98.55 ± 0.58% of Flag co-localized with Tomm20 in MDA-MB-231). Our co-immunoprecipitation experiments showed MTS-Src-Flag binding to the above Complex I subunits (Supplementary Fig. 8).

Since the above data support the role of CDCP1/mitochondrial Src pathway in stimulation of Complex I activity, we assessed the effect of restoration of Complex I activity on OXPHOS in TNBC cells where CDCP1/mitochondrial Src pathway was inhibited. We took advantage of the yeast NADH dehydrogenase (NDI1) enzyme that substitutes for Complex I in Complex I-deficient cells, restoring ETC [69–71]. First, we overexpressed NDI1 in SUM159 cells and confirmed NDI1’s ability to restore ATP production in the presence of the Complex I inhibitor rotenone (Supplementary Fig. 9). Next, we overexpressed NDI1 in CDCP1 knockout or MTS-Kd-Src-Flag-transfected SUM159 cells and showed that NDI1 rescued OXPHOS (Fig. 5e, Supplementary Fig. 10). Of note, in the overexpression setting GFP expression was comparable in gGFP NDI1-GFP and gCDCP1 NDI1-GFP, besides gGFP/Cas9 being stably expressed. These data support the role of CDCP1/mitochondrial Src pathway in stimulating Complex I activity and OXPHOS in TNBC.

### CDCP1/mitochondrial Src axis stimulates TNBC migration through NAD+-generating function of Complex I

**Fig. 6:**
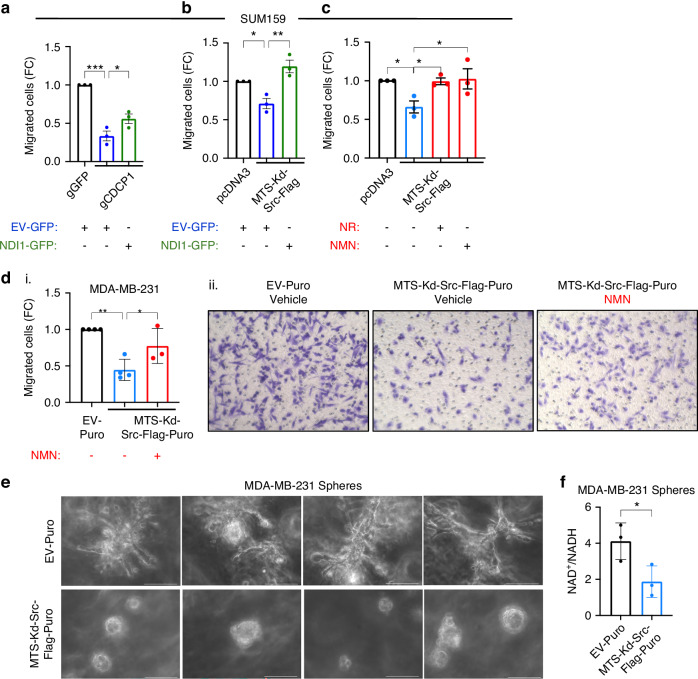
CDCP1/mitochondrial Src axis stimulates TNBC migration and invasion through NAD^+^-generating function of Complex I. **a** NDI1 overexpression partially rescues migration in CDCP1 knockout SUM159 cells (*n* = 3). **b** NDI1 overexpression rescues migration in MTS-Kd-Src-Flag SUM159 cells (*n* = 3). **c** NAD^+^ boosters Nicotinamide Riboside (NR) and Nicotinamide mononucleotide (NMN) rescue migration in MTS-Kd-Src-Flag SUM159 cells (*n* = 3, 0.5 mM NR or NMN treated overnight) and **d** MDA-MB-231 cells (*n* = 3, 0.5 mM NMN treated overnight). All transwell images were taken at 20×, representative images are shown in (ii). **e** Phase contrast images of MDA-MB-231 spheres. (40×) Scale bar = 100 microns. **f** NAD^+^/NADH ratio is significantly reduced in MDA-MB-231 spheres expressing MTS-Kd-Src-Flag-Puro compared to EV-Puro spheres (*n* = 3). *P* values in (**a**–**d**) were analyzed by one way ANOVA with multiple comparison post hoc T-test, error bars represent SEM; *P* values in (**f**) were analyzed by Student’s *t* test, error bars represent SEM; *P < 0.05, **P < 0.01, ***P < 0.001.

The CDCP1/Src signaling axis [14, 55, 58], as well as OXPHOS [5, 7, 8], are known to potentiate migration and metastasis in various disease models. Thus, we aimed to assess the role of CDCP1/mitochondrial Src/Complex I/OXPHOS pathway in TNBC migration. Both knocking out CDCP1 or specifically blocking Src signaling in the mitochondria via MTS-Kd-Src-Flag significantly reduced migration in SUM159 cells, which was rescued by NDI1-mediated restoration of OXPHOS (Fig. 6a, b, Supplementary Fig. 11A, B).

Since one of the functions of Complex I is to oxidize NADH to NAD^+^, and NAD^+^ was reported to stimulate TNBC metastasis in vivo [72], we set to determine if NAD^+^ production is required for CDCP1/Src-mediated cell migration. Importantly, treatment with two different well-characterized NAD^+^ boosters—nicotinamide riboside (NR) and nicotinamide mononucleotide (NMN) [73]—rescued migration in MTS-Kd-Src-Flag cells (Fig. 6c, d and Supplementary Fig. 11C). Because MDA-MB-231 cells are notorious for being highly invasive, we chose this cell line to perform 3D invasion assays. We used lentiviral transduction to stably express either EV-Puro or MTS-Kd-Src-Flag-Puro constructs. Notably, EV-Puro spheres greatly invaded the surrounding 3D matrigel while MTS-Kd-Src-Flag-Puro cells formed perfect spheres that did not invade surrounding matrigel (Fig. 6e, Supplementary Fig. 12A). Lastly, the NAD^+^/NADH ratio was significantly reduced in MTS-Kd-Src-Flag-Puro spheres compared to EV-Puro spheres (Fig. 6f). Similar results were observed in the TNBC line UCI082014 (Supplementary Fig. 12B, C). Thus, NAD^+^ generated by Complex I contributes to TNBC migration and invasiveness.

### CDCP1/Mitochondrial Src inhibition reduces metastasis in MDA-MB-231 cells

**Fig. 7:**
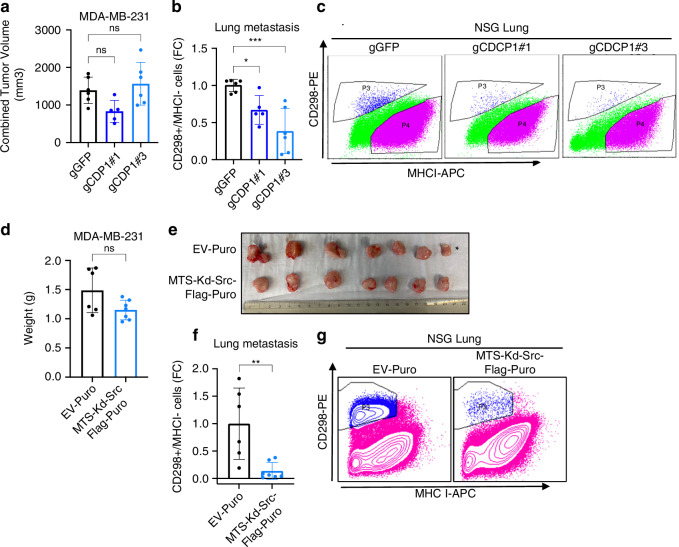
CDCP1/Mitochondrial Src inhibition reduces metastasis in MDA-MB-231 cells. **a** CDCP1 knockout has no effect on combined tumor volume. **b** CDCP1 knockout significantly reduces metastatic spread to the lungs. **c** Representative image of flow cytometry gating strategy. CD298 labels cells of human lineage while MHC-I labels cells of murine lineage. **d** Stable overexpression of MTS-Kd-Src-Flag-Puro has no effect on tumor weight or size. **e** Image of resected tumors. Asterisk denotes tumor that appeared in last week of study and identified as an outlier, excluded from analysis. **f** Stable expression of MTS-Kd-Src-Flag-Puro significantly reduces metastatic spread to the lungs. **g** Representative image of flow cytometry gating strategy. CD298 labels cells of human lineage while MHC-I labels cells of murine lineage. *P* values in (**a**, **b**) were analyzed by one way ANOVA with multiple comparison post hoc T-test, error bars represent SEM; *P* values in (**d**) and (**f**) were analyzed by Student’s *t* test, error bars represent SEM; *P < 0.05, **P < 0.01, ***P < 0.001.

We observed that CDCP1/mitochondrial Src/Complex I pathway promotes migration and invasion in TNBC cells. To determine if this signaling pathway potentiates metastasis in vivo, we orthotopically and bilaterally implanted MDA-MB-231 gGFP or CDCP1 knockout cells into the mammary fat pads of female NSG mice. We observed no significant change in combined tumor volume, but lung metastases were significantly reduced in mice implanted with CDCP1 knockout cells compared to mice with gGFP cells (Fig. 7a–c, Supplementary Fig. 13A). Additionally, we injected MDA-MB-231 cells stably expressing EV-Puro or MTS-Kd-Src-Flag-Puro into the mammary pads of female NSG mice. We observed no significant change in tumor weight between EV-Puro and MTS-Kd-Src-Flag-Puro groups at the end of the study (Fig. 7d, e, Supplementary Fig. 13B). However, lung metastases were significantly reduced in MTS-Kd-Src-Flag-Puro compared to EV-Puro as determined by flow cytometry (Fig. 7f, g). Thus, inhibition of CDCP1/mitochondrial Src signaling axis reduces metastasis in TNBC cells.

Together, our data establish a critical role of CDCP1/mitochondrial Src/Complex I pathway in TNBC metastasis. Thus, both CDCP1 and mitochondrial Src represent potential therapeutic targets to inhibit OXPHOS-mediated TNBC metastasis.

## Discussion

Our studies have elucidated a novel mechanism of metabolic switch towards OXPHOS in TNBC. Specifically, CDCP1 activates Src kinase in mitochondria, which increases activity of ETC, especially Complex I. Importantly, our data show that this novel CDCP1/mitochondrial Src/Complex I signaling axis promotes TNBC metastasis.

Our study shows that CDCP1 signaling is required for phosphorylation of mitochondrial Src, but the exact mechanism of this phosphorylation event remains to be investigated. Super-resolution 3D microscopy allowed us to quantitate the mitochondrial localization of endogenous Src to roughly 20% of total Src volume in TNBC cells, which has not been previously reported. Our data further show that CDCP1 does not localize in mitochondria. Thus, the most plausible mechanism would involve the well documented CDCP1/Src complex on the membrane, leading to CDCP1-dependent Src phosphorylation [14, 55, 56, 58], followed by phospho-Src shuttling to mitochondria. Moreover, the mechanism of Src’s mitochondrial import remains unknown. Accordingly, our study relied on genetically engineered MTS-fused mitochondrially localized Src. Several studies suggest that since Src lacks an MTS, a helper chaperone or adaptor protein is required for mitochondrial import. For example, it is reported that A-kinase anchor protein 12 (AKAP12) recruits and tethers Src to the mitochondria [37], but the precise mechanism for intra-mitochondrial Src import is still to be established, and will likely involve the TOM/TIM import pathway [74].

Importantly, both Src and CDCP1 have developed inhibitors. Dasatinib inhibits multiple kinases including Src family [75], while Saracatinib is more selective towards Src family kinases [76]. However, both Dasatinib and Saracatinib clinical trials had limited effectiveness as single agents in metastatic TNBC patients and metastatic ER^-^/PR^-^ breast cancer patients, respectively [77, 78]. Thus, patient stratification is required as well as combinations with chemotherapy to increase effectiveness. Recent developments in Src family inhibition provide a specific inhibitor with dual action. Besides blocking Src kinase activity (similar to dasatinib and saracatinib), eCF506 locks Src in its inactive conformation abrogating its scaffolding functions [21]. Clinical trials are currently ongoing (NCT05873686). CDCP1 inhibitors work by either inhibiting CDCP1 proteolysis, homodimerization or by forcing its degradation [11, 57, 79]. Importantly, the tumor microenvironment induces CDCP1 activation via cleavage of its extracellular domain, revealing an epitope that can be targeted therapeutically. The antibody specific for cleaved CDCP1 awaits to be tested in the clinic [79]. Since we have shown that CDCP1/Src pathway is active in ~30% of TNBC patient tumors [80], this group of patients represents candidates for blocking this pathway therapeutically.

Several studies have demonstrated that inhibiting OXPHOS in TNBC significantly reduces primary tumor dissemination and metastatic seeding in vivo [5, 7, 8, 12]. Specifically, ATP production is important in driving TNBC metastasis [5, 7, 8], but it is unclear if other metabolites produced from OXPHOS are essential. Our study provides evidence that Complex I-generated NAD^+^ is important for TNBC migration. NAD^+^ is an important coenzyme for NAD^+^-dependent enzymes such as the sirtuin family which regulate cell metabolism, stemness and DNA repair and the poly ADP ribose polymerase (PARP) family of enzymes that maintain genomic stability [81]. NAD^+^ is also a metabolic precursor for NADP^+^/NADPH signaling molecules [82, 83]. Recently, several studies have discovered that NAD^+^ metabolism promotes tumor progression and metastasis in various cancer types including pancreatic, glioblastoma, colon, colorectal and ovarian (reviewed in ref. [81]) [84–88]. Our study is also in line with two recent reports showing that an NAD^+^ booster stimulates TNBC metastasis in vivo [72] and that TNBC metastasis is driven in part by NAD^+^ that has been converted to NADP(H) via NAD^+^ kinase [89], highlighting the pro-metastatic role of NAD^+^ in TNBC. On the other hand, NAD^+^ boosters have been shown to reverse hallmark signs of cellular aging, and these supplements are commercially available [90]. Thus, in light of the above discussion of pro-metastatic functions of NAD^+^ boosters, caution should be executed while taking these supplements.

The central finding of this study is that CDCP1 activates Src kinase in the mitochondria which elevates OXPHOS in TNBC. In this study we focused on the effects of CDCP1/mitochondrial Src axis on Complex I of ETC due to multiple reported Src targets in Complex I [28, 31, 33], as well as our ability to rescue OXPHOS with NDI1 overexpression when CDCP1/Src axis was inhibited. The effect of mitochondrial Src on other ETC complexes remains to be investigated, especially in the context of several reported Src targets in complexes II, IV, and V [28, 30, 32, 34]. Src involvement in metabolism is complex (reviewed in ref. [22]). On one hand, Src regulates glycolysis [23–26, 91], on another hand, it regulates OXPHOS when localized in mitochondrial matrix, where it phosphorylates and/or interacts with multiple proteins involved in OXPHOS, FAO and mitochondrial dynamics [22, 27–32]. Since Src target proteins in mitochondria are largely proposed based on Src interactome and not phosphoproteome [29, 31, 34], with just a few studies reporting the phosphorylation sites [28, 33], and even fewer studies reporting how these phosphorylation events affect mitochondrial protein’s activity [33], our knowledge of mitochondrial Src activity is very limited. In addition, whether these phosphorylation events are increasing [28–34] or decreasing [92] OXPHOS depends on the experimental system and appears to be cell type specific. Specific to TNBC, mitochondrial Src may operate in a “Goldilocks” zone where an endogenous physiologic level of mitochondrial Src promotes OXPHOS but a high supraphysiological level may be deleterious to mitochondrial function [35]. Thus, further investigation into the mechanism needs to be conducted.

The central finding of this study is that CDCP1 activates Src kinase in the mitochondria which elevates OXPHOS in TNBC. In this study we focused on the effects of CDCP1/mitochondrial Src axis on Complex I of ETC due to multiple reported Src targets in Complex I [28, 31, 33], as well as our ability to rescue OXPHOS with NDI1 overexpression when CDCP1/Src axis was inhibited. The effect of mitochondrial Src on other ETC complexes remains to be investigated, especially in the context of several reported Src targets in complexes II, IV, and V [28, 30, 32, 34]. Additionally, the effect of mitochondrial Src on ETC activity seems to be dependent on the model system tested [31, 33, 34, 92]. Specific to TNBC, mitochondrial Src may operate in a “Goldilocks” zone where an endogenous physiologic level of mitochondrial Src promotes OXPHOS but a high supraphysiological level may be deleterious to mitochondrial function [35]. Thus, further investigation into the mechanism needs to be conducted.

OXPHOS is a key driver on TNBC metastasis. However, targeting OXPHOS directly with electron transport chain inhibitors proved to be challenging due to normal tissue toxicity [93]. Thus, targeting TNBC-specific pathways driving OXPHOS represents an advanced strategy to improve therapeutic window. Our findings provide grounds for targeting OXPHOS through inhibiting CDCP1/mitochondrial Src signaling, and position mitochondrial-localized Src as a therapeutic target. It will be important to validate our findings in other CDCP1/Src-driven cancer types that are also reliant on OXPHOS for disease progression such as prostate, melanoma, and colorectal, amongst others [4, 5, 79, 94–97]. To this end, mitochondrial Src may be specifically inhibited via mitochondrial targeted delivery of Src inhibitors. Mitochondrial delivery of other drugs via blended nanoparticle technology was already shown to be feasible [98]. Future studies are required to determine if mitochondrial Src inhibition has therapeutic potential.

## Supplementary information

Supplemental Material

## Data Availability

The data that supports the findings of this study are available from the corresponding author upon reasonable request.

## References

[1] Siegel RL, Miller KD, Fuchs HE, Jemal A. Cancer statistics, 2022. CA Cancer J Clin 2022;72:7–33. 35020204 10.3322/caac.21708

[2] Fahad Ullah M. Breast cancer: current perspectives on the disease status. Adv Exp Med Biol 2019;1152:51–64. 31456179 10.1007/978-3-030-20301-6_4

[3] Siegel RL, Miller KD, Jemal A. Cancer statistics, 2020. CA Cancer J Clin 2020;70:7–30. 31912902 10.3322/caac.21590

[4] Faubert B, Solmonson A, DeBerardinis RJ. Metabolic reprogramming and cancer progression. Science. 2020;368:eaaw5473. 10.1126/science.aaw5473 PMC7227780 32273439

[5] LeBleu VS, O’Connell JT, Gonzalez Herrera KN, Wikman H, Pantel K, Haigis MC, et al. PGC-1alpha mediates mitochondrial biogenesis and oxidative phosphorylation in cancer cells to promote metastasis. Nat Cell Biol 2014;16:992–1003. 25241037 10.1038/ncb3039 PMC4369153

[6] Park JH, Vithayathil S, Kumar S, Sung PL, Dobrolecki LE, Putluri V, et al. Fatty acid oxidation-driven src links mitochondrial energy reprogramming and oncogenic properties in triple-negative breast cancer. Cell Rep. 2016;14:2154–65. 26923594 10.1016/j.celrep.2016.02.004 PMC4809061

[7] Davis RT, Blake K, Ma D, Gabra MBI, Hernandez GA, Phung AT, et al. Transcriptional diversity and bioenergetic shift in human breast cancer metastasis revealed by single-cell RNA sequencing. Nat Cell Biol. 2020;22:310–20. 32144411 10.1038/s41556-020-0477-0

[8] Pacheco-Velazquez SC, Robledo-Cadena DX, Hernandez-Resendiz I, Gallardo-Perez JC, Moreno-Sanchez R, Rodriguez-Enriquez S. Energy metabolism drugs block triple negative breast metastatic cancer cell phenotype. Mol Pharm. 2018;15:2151–64. 29746779 10.1021/acs.molpharmaceut.8b00015

[9] Li YJ, Fahrmann JF, Aftabizadeh M, Zhao Q, Tripathi SC, Zhang C, et al. Fatty acid oxidation protects cancer cells from apoptosis by increasing mitochondrial membrane lipids. Cell Rep. 2022;39:110870. 35649368 10.1016/j.celrep.2022.110870

[10] Ma D, Hernandez GA, Lefebvre A, Alshetaiwi H, Blake K, Dave KR, et al. Patient-derived xenograft culture-transplant system for investigation of human breast cancer metastasis. Commun Biol. 2021;4:1268. 34741115 10.1038/s42003-021-02596-y PMC8571269

[11] Wright HJ, Arulmoli J, Motazedi M, Nelson LJ, Heinemann FS, Flanagan LA, et al. CDCP1 cleavage is necessary for homodimerization-induced migration of triple-negative breast cancer. Oncogene. 2016;35:4762–72. 26876198 10.1038/onc.2016.7 PMC4985505

[12] Wright HJ, Hou J, Xu B, Cortez M, Potma EO, Tromberg BJ, et al. CDCP1 drives triple-negative breast cancer metastasis through reduction of lipid-droplet abundance and stimulation of fatty acid oxidation. Proc Natl Acad Sci USA. 2017;114:E6556–E65. 28739932 10.1073/pnas.1703791114 PMC5559020

[13] Leroy C, Shen Q, Strande V, Meyer R, McLaughlin ME, Lezan E, et al. CUB-domain-containing protein 1 overexpression in solid cancers promotes cancer cell growth by activating Src family kinases. Oncogene. 2015;34:5593–8. 25728678 10.1038/onc.2015.19 PMC4761645

[14] Liu H, Ong SE, Badu-Nkansah K, Schindler J, White FM, Hynes RO. CUB-domain-containing protein 1 (CDCP1) activates Src to promote melanoma metastasis. Proc Natl Acad Sci USA. 2011;108:1379–84. 21220330 10.1073/pnas.1017228108 PMC3029734

[15] Kawase N, Sugihara A, Kajiwara K, Hiroshima M, Akamatsu K, Nada S, et al. SRC kinase activator CDCP1 promotes hepatocyte growth factor-induced cell migration/invasion of a subset of breast cancer cells. J Biol Chem. 2022;298:101630. 35085554 10.1016/j.jbc.2022.101630 PMC8867115

[16] Law ME, Ferreira RB, Davis BJ, Higgins PJ, Kim JS, Castellano RK, et al. CUB domain-containing protein 1 and the epidermal growth factor receptor cooperate to induce cell detachment. Breast Cancer Res. 2016;18:80. 27495374 10.1186/s13058-016-0741-1 PMC4974783

[17] Casar B, He Y, Iconomou M, Hooper JD, Quigley JP, Deryugina EI. Blocking of CDCP1 cleavage in vivo prevents Akt-dependent survival and inhibits metastatic colonization through PARP1-mediated apoptosis of cancer cells. Oncogene. 2012;31:3924–38. 22179830 10.1038/onc.2011.555 PMC4350937

[18] He Y, Davies CM, Harrington BS, Hellmers L, Sheng Y, Broomfield A, et al. CDCP1 enhances Wnt signaling in colorectal cancer promoting nuclear localization of beta-catenin and E-cadherin. Oncogene. 2020;39:219–33. 31471585 10.1038/s41388-019-0983-3

[19] Casar B, Rimann I, Kato H, Shattil SJ, Quigley JP, Deryugina EI. In vivo cleaved CDCP1 promotes early tumor dissemination via complexing with activated beta1 integrin and induction of FAK/PI3K/Akt motility signaling. Oncogene. 2014;33:255–68. 23208492 10.1038/onc.2012.547 PMC3931462

[20] Nandi I, Smith HW, Sanguin-Gendreau V, Ji L, Pacis A, Papavasiliou V, et al. Coordinated activation of c-Src and FOXM1 drives tumor cell proliferation and breast cancer progression. J Clin Invest. 2023;133:e162324. 10.1172/JCI162324 PMC10065076 36795481

[21] Temps C, Lietha D, Webb ER, Li XF, Dawson JC, Muir M, et al. A conformation selective mode of inhibiting SRC improves drug efficacy and tolerability. Cancer Res. 2021;81:5438–50. 34417202 10.1158/0008-5472.CAN-21-0613 PMC7611940

[22] Hebert-Chatelain E. Src kinases are important regulators of mitochondrial functions. Int J Biochem Cell Biol. 2013;45:90–8. 22951354 10.1016/j.biocel.2012.08.014

[23] Ma H, Zhang J, Zhou L, Wen S, Tang HY, Jiang B, et al. c-Src promotes tumorigenesis and tumor progression by activating PFKFB3. Cell Rep. 2020;30:4235–49.e6. 32209481 10.1016/j.celrep.2020.03.005

[24] Jin L, Chun J, Pan C, Alesi GN, Li D, Magliocca KR, et al. Phosphorylation-mediated activation of LDHA promotes cancer cell invasion and tumour metastasis. Oncogene. 2017;36:3797–806. 28218905 10.1038/onc.2017.6 PMC5501759

[25] Ma H, Zhang F, Zhou L, Cao T, Sun D, Wen S, et al. c-Src facilitates tumorigenesis by phosphorylating and activating G6PD. Oncogene. 2021;40:2567–80. 33686238 10.1038/s41388-021-01673-0

[26] Jain S, Wang X, Chang CC, Ibarra-Drendall C, Wang H, Zhang Q, et al. Src inhibition blocks c-Myc translation and glucose metabolism to prevent the development of breast cancer. Cancer Res. 2015;75:4863–75. 26383165 10.1158/0008-5472.CAN-14-2345 PMC4651709

[27] Lurette O, Guedouari H, Morris JL, Martin-Jimenez R, Robichaud JP, Hamel-Cote G, et al. Mitochondrial matrix-localized Src kinase regulates mitochondrial morphology. Cell Mol Life Sci. 2022;79:327. 35637383 10.1007/s00018-022-04325-y PMC9151517

[28] Guedouari H, Savoie MC, Jean S, Djeungoue-Petga MA, Pichaud N, Hebert-Chatelain E. Multi-omics reveal that c-Src modulates the mitochondrial phosphotyrosine proteome and metabolism according to nutrient availability. Cell Physiol Biochem. 2020;54:517–37. 32428391 10.33594/000000237

[29] Guedouari H, Ould Amer Y, Pichaud N, Hebert-Chatelain E. Characterization of the interactome of c-Src within the mitochondrial matrix by proximity-dependent biotin identification. Mitochondrion. 2021;57:257–69. 33412331 10.1016/j.mito.2020.12.012

[30] Arachiche A, Augereau O, Decossas M, Pertuiset C, Gontier E, Letellier T, et al. Localization of PTP-1B, SHP-2, and Src exclusively in rat brain mitochondria and functional consequences. J Biol Chem. 2008;283:24406–11. 18583343 10.1074/jbc.M709217200 PMC3259839

[31] Hebert-Chatelain E, Jose C, Gutierrez Cortes N, Dupuy JW, Rocher C, Dachary-Prigent J, et al. Preservation of NADH ubiquinone-oxidoreductase activity by Src kinase-mediated phosphorylation of NDUFB10. Biochim Biophys Acta. 2012;1817:718–25. 22321370 10.1016/j.bbabio.2012.01.014

[32] Miyazaki T, Neff L, Tanaka S, Horne WC, Baron R. Regulation of cytochrome c oxidase activity by c-Src in osteoclasts. J Cell Biol. 2003;160:709–18. 12615910 10.1083/jcb.200209098 PMC2173369

[33] Ogura M, Yamaki J, Homma MK, Homma Y. Mitochondrial c-Src regulates cell survival through phosphorylation of respiratory chain components. Biochem J. 2012;447:281–9. 22823520 10.1042/BJ20120509 PMC3459221

[34] Hebert Chatelain E, Dupuy JW, Letellier T, Dachary-Prigent J. Functional impact of PTP1B-mediated Src regulation on oxidative phosphorylation in rat brain mitochondria. Cell Mol Life Sci. 2011;68:2603–13. 21063895 10.1007/s00018-010-0573-6 PMC11115002

[35] Djeungoue-Petga MA, Lurette O, Jean S, Hamel-Cote G, Martin-Jimenez R, Bou M, et al. Intramitochondrial Src kinase links mitochondrial dysfunctions and aggressiveness of breast cancer cells. Cell Death Dis. 2019;10:940. 31819039 10.1038/s41419-019-2134-8 PMC6901437

[36] Miyazaki T, Tanaka S, Sanjay A, Baron R. The role of c-Src kinase in the regulation of osteoclast function. Mod Rheumatol. 2006;16:68–74. 16633924 10.1007/s10165-006-0460-z

[37] Livigni A, Scorziello A, Agnese S, Adornetto A, Carlucci A, Garbi C, et al. Mitochondrial AKAP121 links cAMP and src signaling to oxidative metabolism. Mol Biol Cell. 2006;17:263–71. 16251349 10.1091/mbc.E05-09-0827 PMC1345664

[38] Nie K, Li J, He X, Wang Y, Zhao Q, Du M, et al. COX6B2 drives metabolic reprogramming toward oxidative phosphorylation to promote metastasis in pancreatic ductal cancer cells. Oncogenesis. 2020;9:51. 32415061 10.1038/s41389-020-0231-2 PMC7229118

[39] Wu Y, Zhang X, Wang Z, Zheng W, Cao H, Shen W. Targeting oxidative phosphorylation as an approach for the treatment of ovarian cancer. Front Oncol. 2022;12:971479. 36147929 10.3389/fonc.2022.971479 PMC9486401

[40] Fischer GM, Jalali A, Kircher DA, Lee WC, McQuade JL, Haydu LE, et al. Molecular profiling reveals unique immune and metabolic features of melanoma brain metastases. Cancer Discov. 2019;9:628–45. 30787016 10.1158/2159-8290.CD-18-1489 PMC6497554

[41] de Wet L, Williams A, Gillard M, Kregel S, Lamperis S, Gutgesell LC, et al. SOX2 mediates metabolic reprogramming of prostate cancer cells. Oncogene. 2022;41:1190–202. 35067686 10.1038/s41388-021-02157-x PMC8858874

[42] Beier AK, Puhr M, Stope MB, Thomas C, Erb HHH. Metabolic changes during prostate cancer development and progression. J Cancer Res Clin Oncol. 2023;149:2259–70. 36151426 10.1007/s00432-022-04371-w PMC10097763

[43] Razorenova OV, Ivanov AV, Budanov AV, Chumakov PM. Virus-based reporter systems for monitoring transcriptional activity of hypoxia-inducible factor 1. Gene. 2005;350:89–98. 15794924 10.1016/j.gene.2005.02.006 PMC2773277

[44] Razorenova OV, Castellini L, Colavitti R, Edgington LE, Nicolau M, Huang X, et al. The apoptosis repressor with a CARD domain (ARC) gene is a direct hypoxia-inducible factor 1 target gene and promotes survival and proliferation of VHL-deficient renal cancer cells. Mol Cell Biol. 2014;34:739–51. 24344197 10.1128/MCB.00644-12 PMC3911479

[45] Telford JE, Kilbride SM, Davey GP. Complex I is rate-limiting for oxygen consumption in the nerve terminal. J Biol Chem. 2009;284:9109–14. 19193637 10.1074/jbc.M809101200 PMC2666560

[46] Li N, Ragheb K, Lawler G, Sturgis J, Rajwa B, Melendez JA, et al. Mitochondrial complex I inhibitor rotenone induces apoptosis through enhancing mitochondrial reactive oxygen species production. J Biol Chem. 2003;278:8516–25. 12496265 10.1074/jbc.M210432200

[47] Tieu K, Perier C, Caspersen C, Teismann P, Wu DC, Yan SD, et al. D-beta-hydroxybutyrate rescues mitochondrial respiration and mitigates features of Parkinson disease. J Clin Invest. 2003;112:892–901. 12975474 10.1172/JCI18797 PMC193668

[48] Choi WS, Kruse SE, Palmiter RD, Xia Z. Mitochondrial complex I inhibition is not required for dopaminergic neuron death induced by rotenone, MPP+, or paraquat. Proc Natl Acad Sci USA. 2008;105:15136–41. 18812510 10.1073/pnas.0807581105 PMC2567505

[49] Stringari C, Nourse JL, Flanagan LA, Gratton E. Phasor fluorescence lifetime microscopy of free and protein-bound NADH reveals neural stem cell differentiation potential. PLoS One. 2012;7:e48014. 23144844 10.1371/journal.pone.0048014 PMC3489895

[50] Claude A. Fractionation of mammalian liver cells by differential centrifugation: I. Problems, methods, and preparation of extract. J Exp Med. 1946;84:51–9. 19871553 PMC2135638

[51] Claude A, Fullam EF. An electron microscope study of isolated mitochondria: method and preliminary results. J Exp Med 1945;81:51–62. 19871443 10.1084/jem.81.1.51 PMC2135528

[52] Zhang Y, Cao F, Zhou Y, Feng Z, Sit B, Krendel M, et al. Tail domains of myosin-1e regulate phosphatidylinositol signaling and F-actin polymerization at the ventral layer of podosomes. Mol Biol Cell. 2019;30:622–35. 30601698 10.1091/mbc.E18-06-0398 PMC6589698

[53] Cannino G, El-Khoury R, Pirinen M, Hutz B, Rustin P, Jacobs HT, et al. Glucose modulates respiratory complex I activity in response to acute mitochondrial dysfunction. J Biol Chem. 2012;287:38729–40. 23007390 10.1074/jbc.M112.386060 PMC3493916

[54] Lawson DA, Bhakta NR, Kessenbrock K, Prummel KD, Yu Y, Takai K, et al. Single-cell analysis reveals a stem-cell program in human metastatic breast cancer cells. Nature. 2015;526:131–5. 26416748 10.1038/nature15260 PMC4648562

[55] Kajiwara K, Yamano S, Aoki K, Okuzaki D, Matsumoto K, Okada M. CDCP1 promotes compensatory renal growth by integrating Src and Met signaling. Life Sci Alliance. 2021;4:e202000832. 10.26508/lsa.202000832 PMC7893822 33574034

[56] Kollmorgen G, Bossenmaier B, Niederfellner G, Haring HU, Lammers R. Structural requirements for cub domain containing protein 1 (CDCP1) and Src dependent cell transformation. PLoS One. 2012;7:e53050. 23300860 10.1371/journal.pone.0053050 PMC3534080

[57] Kollmorgen G, Niederfellner G, Lifke A, Spohn GJ, Rieder N, Harring SV, et al. Antibody mediated CDCP1 degradation as mode of action for cancer targeted therapy. Mol Oncol. 2013;7:1142–51. 24055141 10.1016/j.molonc.2013.08.009 PMC5528444

[58] Kajiwara K, Chen PK, Abe Y, Okuda S, Kon S, Adachi J, et al. Src activation in lipid rafts confers epithelial cells with invasive potential to escape from apical extrusion during cell competition. Curr Biol. 2022;32:3460–76.e6. 35809567 10.1016/j.cub.2022.06.038

[59] Bhatt AS, Erdjument-Bromage H, Tempst P, Craik CS, Moasser MM. Adhesion signaling by a novel mitotic substrate of src kinases. Oncogene. 2005;24:5333–43. 16007225 10.1038/sj.onc.1208582 PMC3023961

[60] Drozdowicz-Tomsia K, Anwer AG, Cahill MA, Madlum KN, Maki AM, Baker MS, et al. Multiphoton fluorescence lifetime imaging microscopy reveals free-to-bound NADH ratio changes associated with metabolic inhibition. J Biomed Opt. 2014;19:086016. 25140884 10.1117/1.JBO.19.8.086016

[61] He Y, Wortmann A, Burke LJ, Reid JC, Adams MN, Abdul-Jabbar I, et al. Proteolysis-induced N-terminal ectodomain shedding of the integral membrane glycoprotein CUB domain-containing protein 1 (CDCP1) is accompanied by tyrosine phosphorylation of its C-terminal domain and recruitment of Src and PKCdelta. J Biol Chem. 2010;285:26162–73. 20551327 10.1074/jbc.M109.096453 PMC2924022

[62] Donepudi M, Resh MD. c-Src trafficking and co-localization with the EGF receptor promotes EGF ligand-independent EGF receptor activation and signaling. Cell Sig. 2008;20:1359–67. 10.1016/j.cellsig.2008.03.007 PMC2459337 18448311

[63] Murphy MP. How mitochondria produce reactive oxygen species. Biochem J. 2009;417:1–13. 19061483 10.1042/BJ20081386 PMC2605959

[64] Brand MD. The sites and topology of mitochondrial superoxide production. Exp Gerontol. 2010;45:466–72. 20064600 10.1016/j.exger.2010.01.003 PMC2879443

[65] Rodriguez-Nuevo A, Torres-Sanchez A, Duran JM, De Guirior C, Martinez-Zamora MA, Boke E. Oocytes maintain ROS-free mitochondrial metabolism by suppressing complex I. Nature. 2022;607:756–61. 35859172 10.1038/s41586-022-04979-5 PMC9329100

[66] Fato R, Bergamini C, Bortolus M, Maniero AL, Leoni S, Ohnishi T, et al. Differential effects of mitochondrial Complex I inhibitors on production of reactive oxygen species. Biochim Biophys Acta. 2009;1787:384–92. 19059197 10.1016/j.bbabio.2008.11.003 PMC2724837

[67] Kauffman ME, Kauffman MK, Traore K, Zhu H, Trush MA, Jia Z, et al. MitoSOX-based flow cytometry for detecting mitochondrial ROS. React Oxyg Species. 2016;2:361–70. 10.20455/ros.2016.865 PMC5926237 29721549

[68] Ge H, Zhao M, Lee S, Xu Z. Mitochondrial Src tyrosine kinase plays a role in the cardioprotective effect of ischemic preconditioning by modulating complex I activity and mitochondrial ROS generation. Free Radic Res. 2015;49:1210–7. 25968938 10.3109/10715762.2015.1050013

[69] Li H, Sun B, Huang Y, Zhang J, Xu X, Shen Y, et al. Gene therapy of yeast NDI1 on mitochondrial complex I dysfunction in rotenone-induced Parkinson’s disease models in vitro and vivo. Mol Med. 2022;28:29. 35255803 10.1186/s10020-022-00456-x PMC8900322

[70] McElroy GS, Reczek CR, Reyfman PA, Mithal DS, Horbinski CM, Chandel NS. NAD+ regeneration rescues lifespan, but not ataxia, in a mouse model of brain mitochondrial complex I dysfunction. Cell Metab. 2020;32:301–8.e6. 32574562 10.1016/j.cmet.2020.06.003 PMC7415718

[71] Billingham LK, Stoolman JS, Vasan K, Rodriguez AE, Poor TA, Szibor M, et al. Mitochondrial electron transport chain is necessary for NLRP3 inflammasome activation. Nat Immunol. 2022;23:692–704. 35484407 10.1038/s41590-022-01185-3 PMC9098388

[72] Maric T, Bazhin A, Khodakivskyi P, Mikhaylov G, Solodnikova E, Yevtodiyenko A, et al. A bioluminescent-based probe for in vivo non-invasive monitoring of nicotinamide riboside uptake reveals a link between metastasis and NAD(+) metabolism. Biosens Bioelectron. 2023;220:114826. 36371959 10.1016/j.bios.2022.114826

[73] Yoshino J, Baur JA, Imai SI. NAD(+) intermediates: the biology and therapeutic potential of NMN and NR. Cell Metab. 2018;27:513–28. 29249689 10.1016/j.cmet.2017.11.002 PMC5842119

[74] Harbauer AB, Zahedi RP, Sickmann A, Pfanner N, Meisinger C. The protein import machinery of mitochondria-a regulatory hub in metabolism, stress, and disease. Cell Metab. 2014;19:357–72. 24561263 10.1016/j.cmet.2014.01.010

[75] Kim LC, Rix U, Haura EB. Dasatinib in solid tumors. Expert Opin Investig Drugs. 2010;19:415–25. 20113198 10.1517/13543781003592097

[76] Hennequin LF, Allen J, Breed J, Curwen J, Fennell M, Green TP, et al. N-(5-chloro-1,3-benzodioxol-4-yl)-7-[2-(4-methylpiperazin-1-yl)ethoxy]-5- (tetrahydro-2H-pyran-4-yloxy)quinazolin-4-amine, a novel, highly selective, orally available, dual-specific c-Src/Abl kinase inhibitor. J Med Chem. 2006;49:6465–88. 10.1021/jm060434q 17064066

[77] Finn RS, Bengala C, Ibrahim N, Roche H, Sparano J, Strauss LC, et al. Dasatinib as a single agent in triple-negative breast cancer: results of an open-label phase 2 study. Clin Cancer Res. 2011;17:6905–13. 22028489 10.1158/1078-0432.CCR-11-0288

[78] Gucalp A, Sparano JA, Caravelli J, Santamauro J, Patil S, Abbruzzi A, et al. Phase II trial of saracatinib (AZD0530), an oral SRC-inhibitor for the treatment of patients with hormone receptor-negative metastatic breast cancer. Clin Breast Cancer. 2011;11:306–11. 21729667 10.1016/j.clbc.2011.03.021 PMC3222913

[79] Lim SA, Zhou J, Martinko AJ, Wang YH, Filippova EV, Steri V, et al. Targeting a proteolytic neoepitope on CUB domain containing protein 1 (CDCP1) for RAS-driven cancers. J Clin Invest. 2022;132:e154604. 10.1172/JCI154604 PMC8843743 35166238

[80] Nelson LJ, Wright HJ, Dinh NB, Nguyen KD, Razorenova OV, Heinemann FS. Src kinase is biphosphorylated at Y416/Y527 and activates the CUB-domain containing protein 1/protein kinase C delta pathway in a subset of triple-negative breast cancers. Am J Pathol. 2020;190:484–502. 31843498 10.1016/j.ajpath.2019.10.017 PMC6983918

[81] Xie N, Zhang L, Gao W, Huang C, Huber PE, Zhou X, et al. NAD(+) metabolism: pathophysiologic mechanisms and therapeutic potential. Sig Transduct Target Ther. 2020;5:227. 10.1038/s41392-020-00311-7 PMC7539288 33028824

[82] Fang Y, Tang S, Li X. Sirtuins in metabolic and epigenetic regulation of stem cells. Trends Endocrinol Metab. 2019;30:177–88. 30630664 10.1016/j.tem.2018.12.002 PMC6382540

[83] Xiao W, Wang RS, Handy DE, Loscalzo J. NAD(H) and NADP(H) redox couples and cellular energy metabolism. Antioxid Redox Signal. 2018;28:251–72. 28648096 10.1089/ars.2017.7216 PMC5737637

[84] Nacarelli T, Lau L, Fukumoto T, Zundell J, Fatkhutdinov N, Wu S, et al. NAD(+) metabolism governs the proinflammatory senescence-associated secretome. Nat Cell Biol. 2019;21:397–407. 30778219 10.1038/s41556-019-0287-4 PMC6448588

[85] Gujar AD, Le S, Mao DD, Dadey DY, Turski A, Sasaki Y, et al. An NAD+-dependent transcriptional program governs self-renewal and radiation resistance in glioblastoma. Proc Natl Acad Sci USA. 2016;113:E8247–E56. 27930300 10.1073/pnas.1610921114 PMC5187672

[86] Lucena-Cacace A, Otero-Albiol D, Jimenez-Garcia MP, Munoz-Galvan S, Carnero A. NAMPT is a potent oncogene in colon cancer progression that modulates cancer stem cell properties and resistance to therapy through Sirt1 and PARP. Clin Cancer Res. 2018;24:1202–15. 29203587 10.1158/1078-0432.CCR-17-2575

[87] Zhou L, Liu H, Chen Z, Chen S, Lu J, Liu C, et al. Downregulation of miR-182-5p by NFIB promotes NAD+ salvage synthesis in colorectal cancer by targeting NAMPT. Commun Biol. 2023;6:775. 37491379 10.1038/s42003-023-05143-z PMC10368701

[88] Piacente F, Caffa I, Ravera S, Sociali G, Passalacqua M, Vellone VG, et al. Nicotinic acid phosphoribosyltransferase regulates cancer cell metabolism, susceptibility to NAMPT inhibitors, and DNA repair. Cancer Res. 2017;77:3857–69. 28507103 10.1158/0008-5472.CAN-16-3079

[89] Ilter D, Drapela S, Schild T, Ward NP, Adhikari E, Low V, et al. NADK-mediated de novo NADP(H) synthesis is a metabolic adaptation essential for breast cancer metastasis. Redox Biol. 2023;61:102627. 36841051 10.1016/j.redox.2023.102627 PMC9982641

[90] Covarrubias AJ, Perrone R, Grozio A, Verdin E. NAD(+) metabolism and its roles in cellular processes during ageing. Nat Rev Mol Cell Biol. 2021;22:119–41. 33353981 10.1038/s41580-020-00313-x PMC7963035

[91] Zhang J, Wang S, Jiang B, Huang L, Ji Z, Li X, et al. c-Src phosphorylation and activation of hexokinase promotes tumorigenesis and metastasis. Nat Commun. 2017;8:13732. 28054552 10.1038/ncomms13732 PMC5227066

[92] Hunter CA, Koc H, Koc EC. c-Src kinase impairs the expression of mitochondrial OXPHOS complexes in liver cancer. Cell Sig. 2020;72:109651. 10.1016/j.cellsig.2020.109651 PMC9373045 32335258

[93] Yap TA, Daver N, Mahendra M, Zhang J, Kamiya-Matsuoka C, Meric-Bernstam F, et al. Complex I inhibitor of oxidative phosphorylation in advanced solid tumors and acute myeloid leukemia: phase I trials. Nat Med. 2023;29:115–26. 36658425 10.1038/s41591-022-02103-8 PMC11975418

[94] Kumar R, Chaudhary AK, Woytash J, Inigo JR, Gokhale AA, Bshara W, et al. A mitochondrial unfolded protein response inhibitor suppresses prostate cancer growth in mice via HSP60. J Clin Invest. 2022;132:e149906. 10.1172/JCI149906 PMC9246382 35653190

[95] Zheng J, Wang Q, Chen J, Cai G, Zhang Z, Zou H, et al. Tumor mitochondrial oxidative phosphorylation stimulated by the nuclear receptor RORgamma represents an effective therapeutic opportunity in osteosarcoma. Cell Rep Med. 2024;5:101519. 38692271 10.1016/j.xcrm.2024.101519 PMC11148566

[96] Niu N, Shen X, Wang Z, Chen Y, Weng Y, Yu F, et al. Tumor cell-intrinsic epigenetic dysregulation shapes cancer-associated fibroblasts heterogeneity to metabolically support pancreatic cancer. Cancer Cell. 2024;42:869–84.e9. 38579725 10.1016/j.ccell.2024.03.005

[97] Kumar PR, Moore JA, Bowles KM, Rushworth SA, Moncrieff MD. Mitochondrial oxidative phosphorylation in cutaneous melanoma. Br J Cancer. 2021;124:115–23. 33204029 10.1038/s41416-020-01159-y PMC7782830

[98] Marrache S, Dhar S. Engineering of blended nanoparticle platform for delivery of mitochondria-acting therapeutics. Proc Natl Acad Sci USA. 2012;109:16288–93. 22991470 10.1073/pnas.1210096109 PMC3479596

