# Supplementary material for: CDCP1/mitochondrial Src axis increases electron transport chain function to promote metastasis in triple-negative breast cancer
Source: Br J Cancer. 2025 Sep 4;133(9):1265–77. doi: 10.1038/s41416-025-03163-6 (PMC12530134; doi:10.1038/s41416-025-03163-6)
Supplement: Supplementary file 1 — Supplemental Material [file 41416_2025_3163_MOESM1_ESM.pdf]

Supplemental Table 1. Antibodies

Antibody	Dilution	Catalog #	Application
CDCP1	1:2,000	Cell Signaling #13794S	Western Blot
beta-actin	1:5,000	Sigma-Aldrich A5441	Western Blot
p-Src Y416	1:500	Cell Signaling #2101S	Western Blot
Src	1:1,000	Cell Signaling #2109S	Western Blot
p-PKCδ Y311	1:500	Cell Signaling #2055S	Western Blot
PKCδ	1:1,000	Cell Signaling #9616S	Western Blot
Flag	1:500	Cell Signaling #14793S	Western Blot
NDUFB10	1:1,000	Abcam #ab196019	Western Blot
NDUFBA8	1:1,000	Abcam #ab184952	Western Blot
NDUFS1	1:1,000	Abcam #ab169540	Western Blot
Tomm20	1:1,000	Abcam #ab186735	Western Blot
LDHA	1:1,000	Santa Cruz Biotechnology #sc-137243	Western Blot
COXIV	1:1,000	Cell Signaling #4850S	Western Blot
Src	1:100	Cell Signaling #2109S	Immunofluorescence
p-Src Y419	1:100	Thermo Fisher Scientific #44660G	Immunofluorescence
Flag	1:100	Cell Signaling #14793S	Immunofluorescence
Tomm20	1:400	Abcam #ab186735	Immunofluorescence
CDCP1	1:100	Cell Signaling #4115R	Immunofluorescence
APC anti-human CD318 (CDCP1)	5 µL/ 1x10^6 cells	Biolegend #324008	FACS
p-Src Y527	1:500	Cell Signaling #2105S	Western Blot

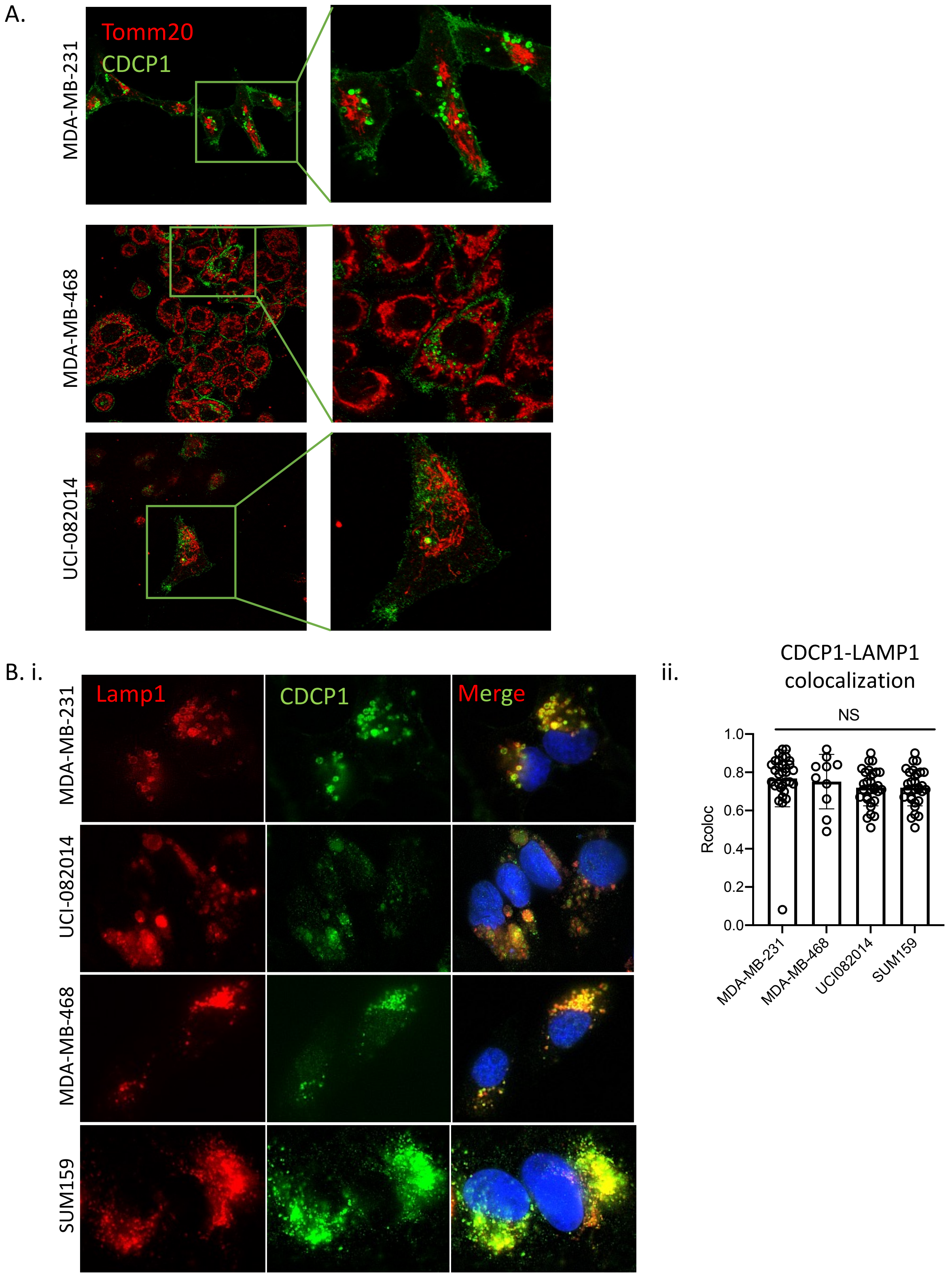

Supplemental Fig. 1 | CDCP1 localizes at the cell membrane and in endosomes, but not in mitochondria in TNBC. A) CDCP1 (green) and Tomm20 (mitochondrial marker, red) localizations were assessed using the ZEISS LSM780 confocal microscope. All images were taken at 63X. Insets are digitally zoomed in for better visualization. **B)** CDCP1 (green) and LAMP1 (late endosome/lysosomal marker **(i)**, red) were assessed using the Keyence BZ-X810 fluorescent microscope at 63X and quantitated using ImageJ's colocalization macro (Rcoloc) **(ii).**

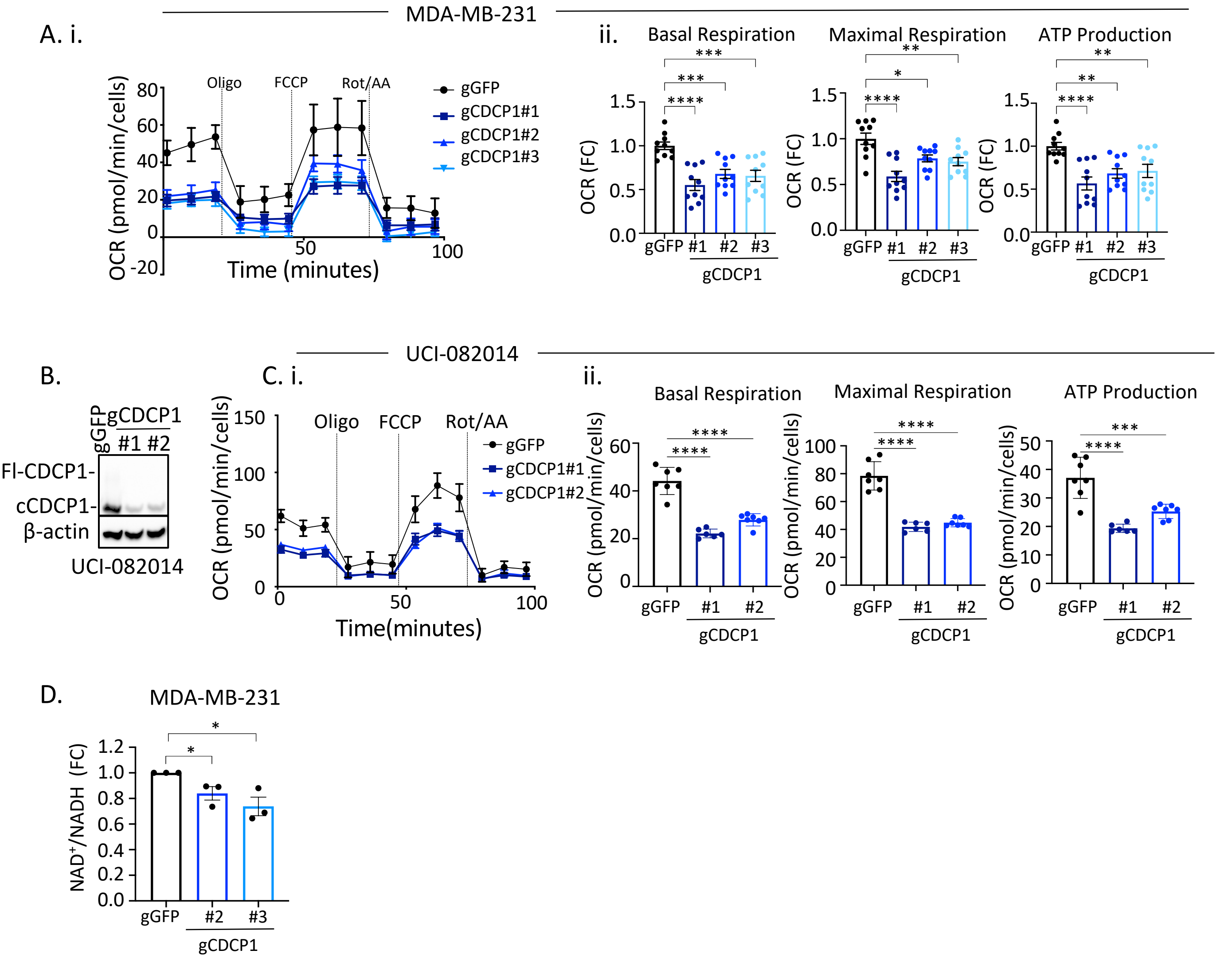

Supplemental Fig. 2 | CDCP1 increases OXPHOS in TNBC cells. Ai. & **Ci.** show representative Seahorse XF oxygen consumption rate (OCR) curves and **Aii** & **Cii.** show quantification. OCR is shown in response to treatments with Oligomycin (Oligo), carbonyl cyanide-p-trifluoromethoxyphenylhydrazone (FCCP), and Rotenone/Antimycin A (Rot/AA). Basal and maximal respiration were determined by Seahorse Mito Stress Test analysis in addition to ATP production. **B.** shows western blot confirming CDCP1 knockout in UCI-082014. Western blot confirming CDCP1 knockout in MDA-MB-231 is shown in **Fig. 1B. D.** depicts change in $NAD^+/NADH$ ratio in CDCP1 knockout MDA-MB-231 cells compared to gGFP control.

Supplemental Fig. 3

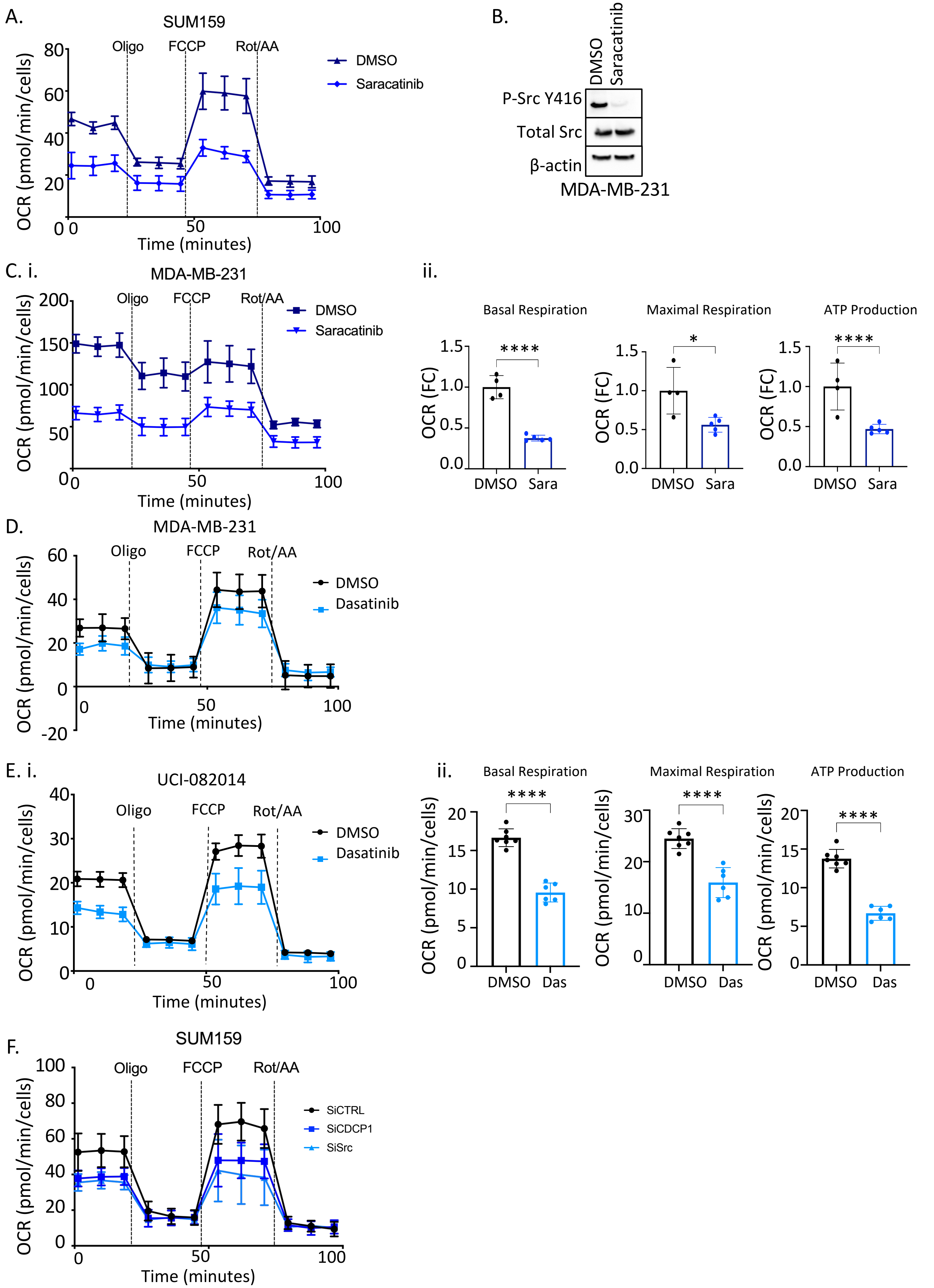

Supplemental Fig. 3 | Src increases OXPHOS in TNBC cells. A) Representative OCR curves for SUM159 cells treated with 1 μM Saracatinib for 16 hours, associated with **Fig. 2B**&**C**. **B)** Western blot showing Src inhibition in MDA-MB-231 cells by treatment with 1 μM Saracatinib for 16h–phospho-Src Y416 is reduced. **C)** Src inhibition by Saracatinib (Sara) treatment reduces OXPHOS in MDA-MB-231 cells. **i)** Representative OCR curves and **ii)** quantitation (n=5 per condition). **D)** Representative OCR curves for MDA-MB-231 cells treated with 10 μM Dasatinib for 16 hours. OCR curves are associated with **Fig. 2D**&**E**. **E)** Src inhibition by Dasatinib (Das) reduces OXPHOS in UCI-082014 cells. **i)** Representative OCR curve and **ii)** quantitation (n=6 per condition). Seahorse experiments were conducted as described in **Fig. 2A**. **F)** Representative OCR curves for SUM159 cells with either CDCP1 or Src transient knockdown by siRNAs, associated with **Fig 2G**&**H**. P values analyzed by student's t-test and error bars represent StDev. *P<0.05, ****P<0.0001.

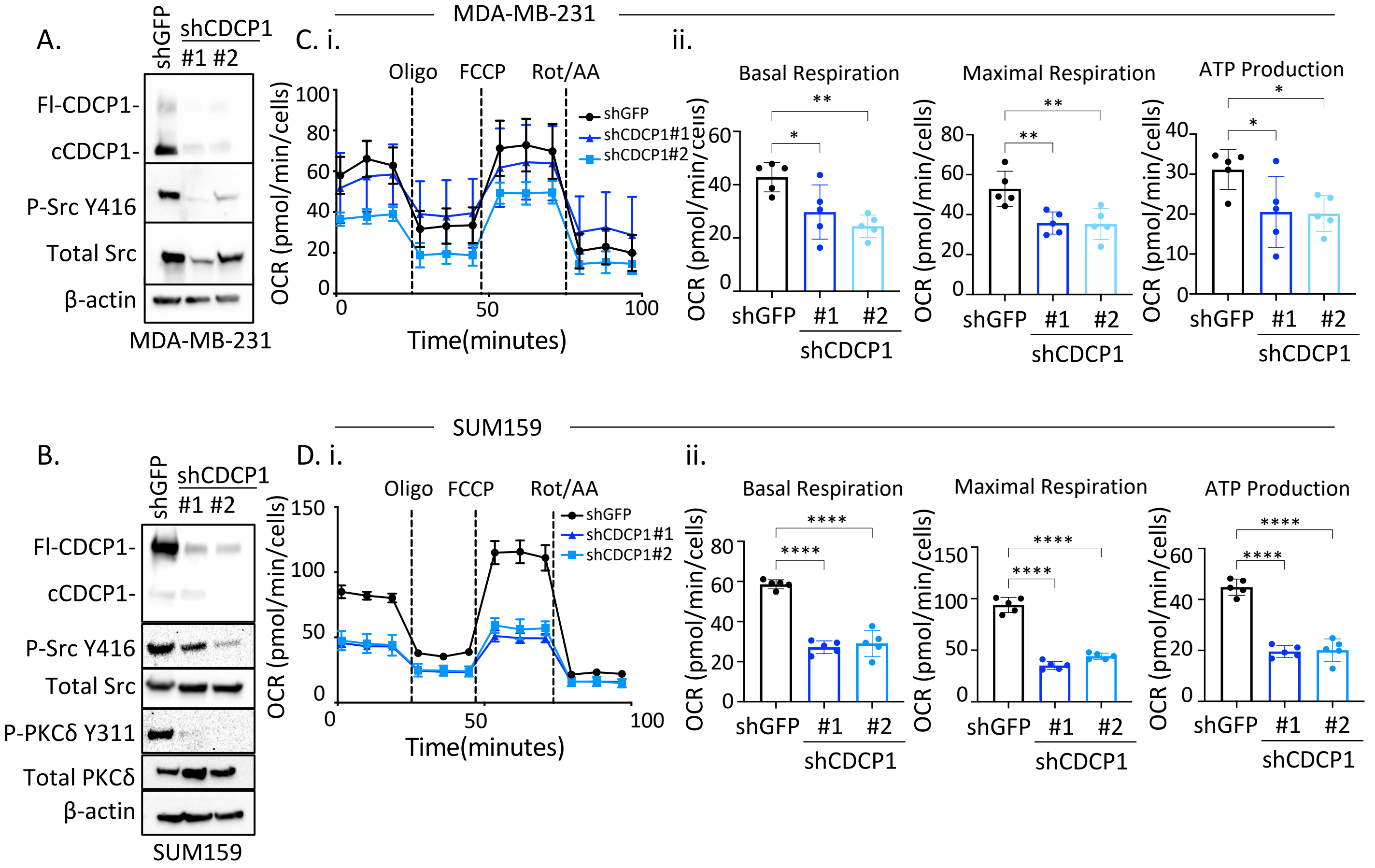

Supplemental Fig. 4 | Stable CDCP1 knockdown reduces OXPHOS in TNBC cells. Stable CDCP1 knockdowns reduce OXPHOS in TNBC cell lines indicated as measured by Seahorse XF24 analyzer. **A-B** shows western blot confirming CDCP1 knockdown and abrogation of Src signaling. **Ci.** & **Di.** show representative oxygen consumption rate (OCR) curves and **Cii.** & **Dii.** show quantification (n=5 per condition). OCR is shown in response to treatments with Oligomycin (Oligo), carbonyl cyanide-p-trifluoromethoxyphenylhydrazone (FCCP), and Rotenone/Antimycin A (Rot/AA). Basal and maximal respiration were determined by Seahorse Mito Stress Test analysis in addition to ATP production. P values analyzed by one way ANOVA with multiple comparison post hoc analysis and error bars represent StDevs and. *P<0.05, **P<0.01, ***P<0.001, ****P<0.0001

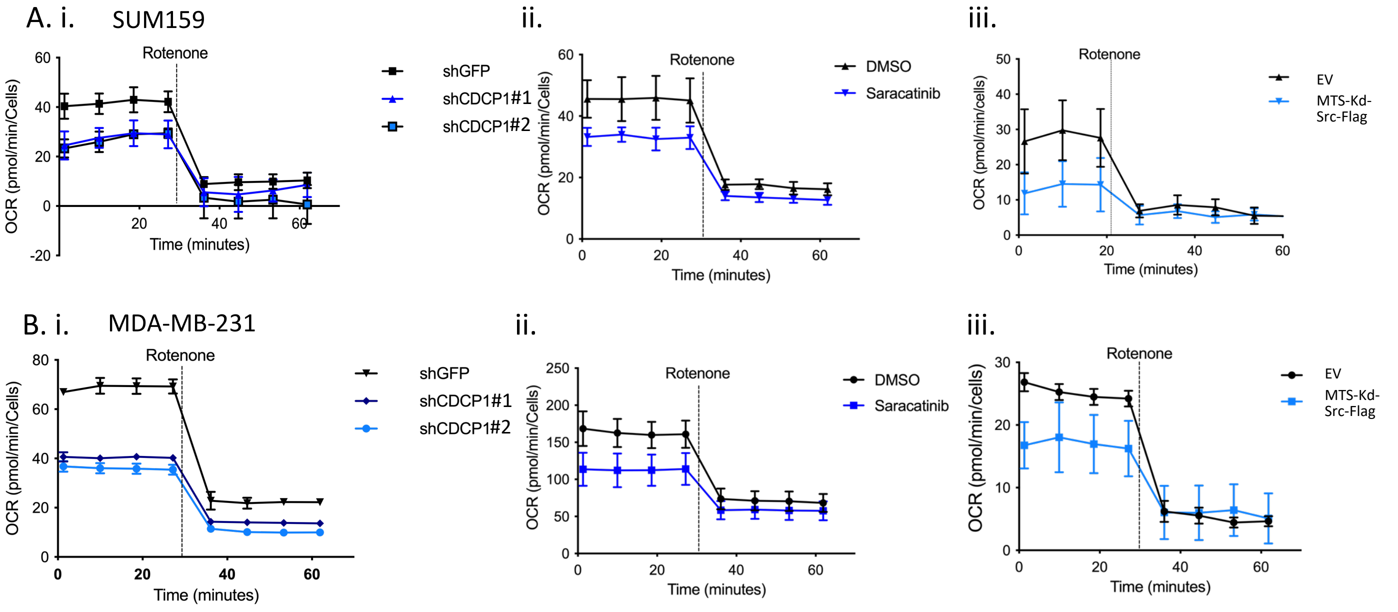

Supplemental Fig. 5 | CDCP1/Src axis inhibition reduces Complex I activity, A-B) Representative Complex I activity curves in CDCP1 knockdown cells **(i)**, Saracatinib-treated cells **(ii)** and mitochondrial-localized kinase-dead Src (MTS-Kd-Src-Flag)-transduced cells **(iii)**. Cell lines as indicated. OCR curves in **A** and **B** are associated with **Fig. 5A** and **C** respectively.

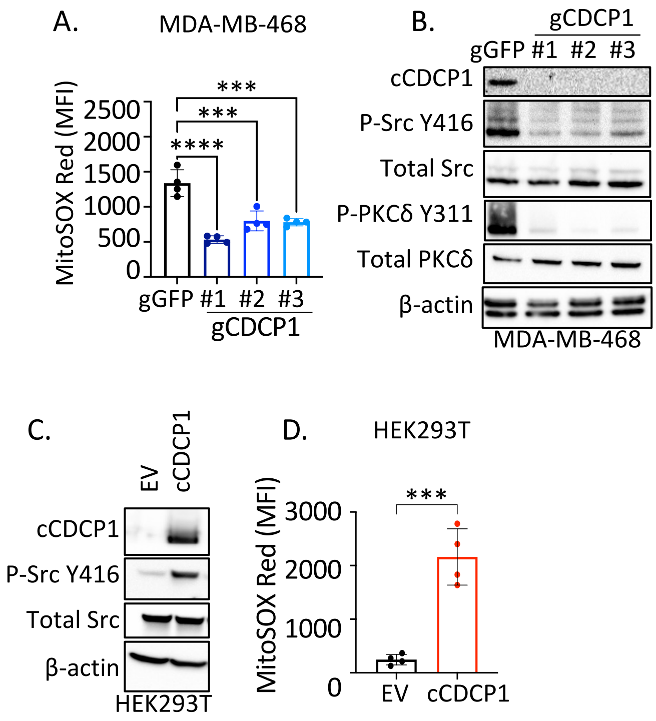

Supplemental Fig. 6 | CDCP1 knockout decreases and cCDCP1 overexpression increases mitochondrial-specific ROS. A) Mitochondrial-specific ROS decreased in MDA-MB-468 cells where CDCP1 was knocked out with CRISPR/Cas9 and two to three different gRNAs. **B)** Western blot confirming CDCP1 knockout in MDA-MB-468 cells with CRISPR/Cas9 and three different gRNAs. CDCP1 knockout led to a decrease in Src and PKCδ (Src substrate) phosphorylation. gRNA targeting GFP was used as a control in **A-B**. **C)** Western blot confirming cCDCP1 overexpression in HEK293T cells, accompanied by the increase in Src phosphorylation. HEK293T were transfected with cleaved active form of CDCP1 (cCDCP1) or empty vector-control (EV) and used for experiments 48h post-transfection. **D)** cCDCP1 overexpression increased mitochondrial-specific ROS production. In **A&D** MitoSOX red staining was quantified via flow cytometry. P values in **A** were analyzed by one way ANOVA with multiple comparison post hoc T-test, while P values in **D** were analyzed by student's t-test; error bars represent SEM; ***$P<0.001$, ****$P<0.0001$.

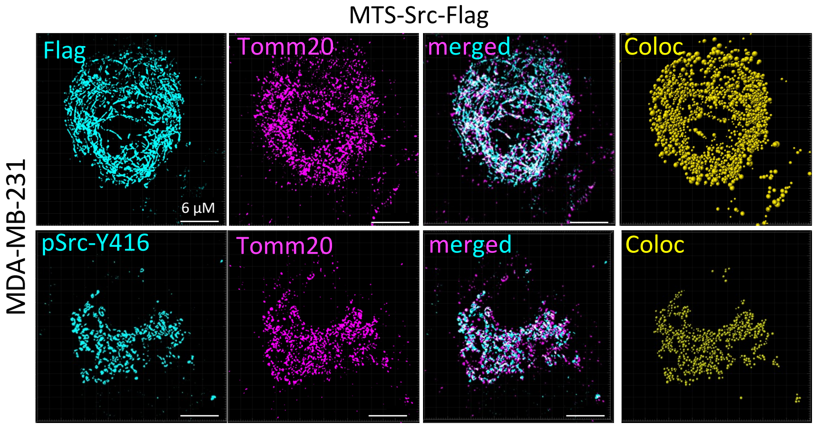

Supplemental Fig. 7 | Immunofluorescent images showing that Flag-tagged mitochondrially-targeted wild-type Src (MTS-Src-Flag) efficiently localizes to mitochondria in MDA-MB-231 cells as assessed by super-resolution microscopy. Immunofluorescent images of Flag (cyan), Tomm20 (magenta), merged images and colocalization (yellow), see **Fig. 3** for details. Scale bar = 6 microns.

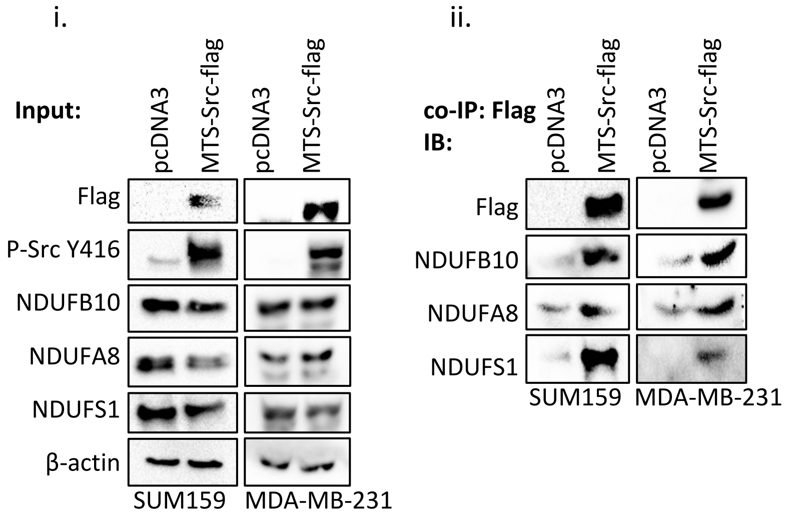

Supplemental Fig. 8 | Mitochondrial Src binds several subunits of Complex I. SUM159 and MDA-MB-231 cells were transfected with Flag-tagged mitochondrial-localized wild-type Src (MTS-Src-Flag). **i)** inputs, **ii)** co-immunoprecipitations (co-IPs): MTS-Src-Flag was pulled down via Flag IP and binding with Complex I subunits was assessed via western blot analysis. Cell lines as indicated.

A.

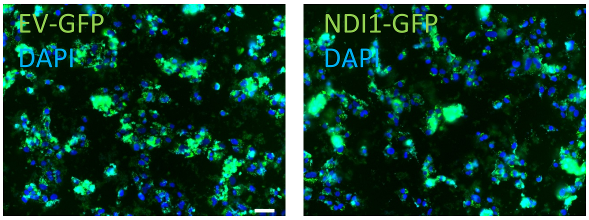

B.

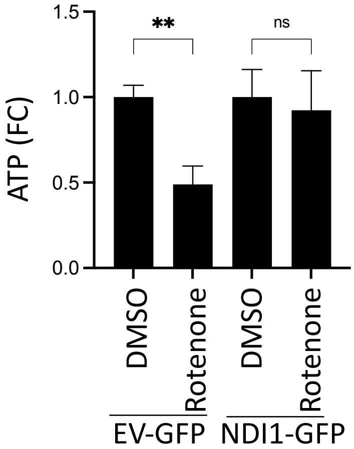

Supplemental Fig. 9 | NDI1 rescues rotenone-induced ATP depletion. A) High transfection efficiency of SUM159 cells with either EV-GFP empty vector control or NDI1-GFP was confirmed via fluorescent microscopy (20x) and estimated to be close to 100%. GFP in NDI1-GFP is expressed via internal ribosome entry site, thus it has cytoplasmic localization. **B)** 48h post-transfection cells were treated with either DMSO or 0.5 μM rotenone for 45 minutes, and then the luminescent ATP detection assay was performed with 4 technical replicates. Data were normalized to DMSO and graphed as ATP fold change (FC). Statistical analysis was conducted by one-way ANOVA with multiple comparison post-hoc T-test and error bars represent StDev. $^{**}P<0.01$.

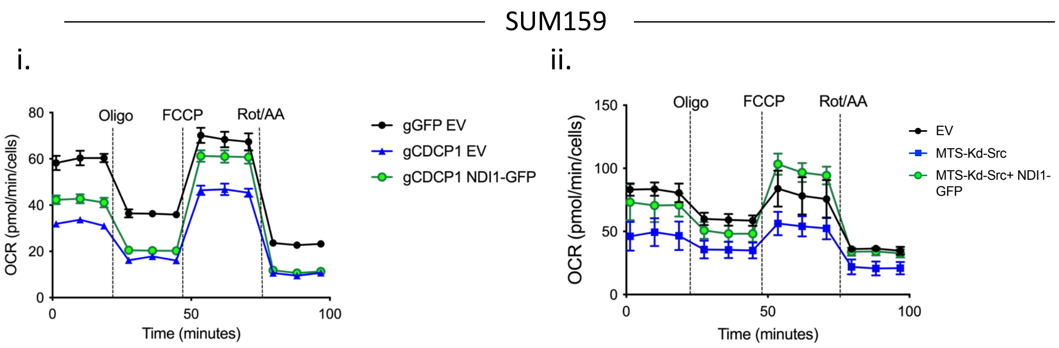

Supplemental Fig. 10 | NDI1 rescues OXPHOS in CDCP1/Src-deficient cells. Representative OCR curves for NDI1 rescue in CDCP1 knockout **(i)** and MTS-Kd-Src-Flag overexpressing **(ii)** SUM159 cells. Seahorse experiments were conducted as described in **Fig. 2A** and associated with **Fig. 5E.**

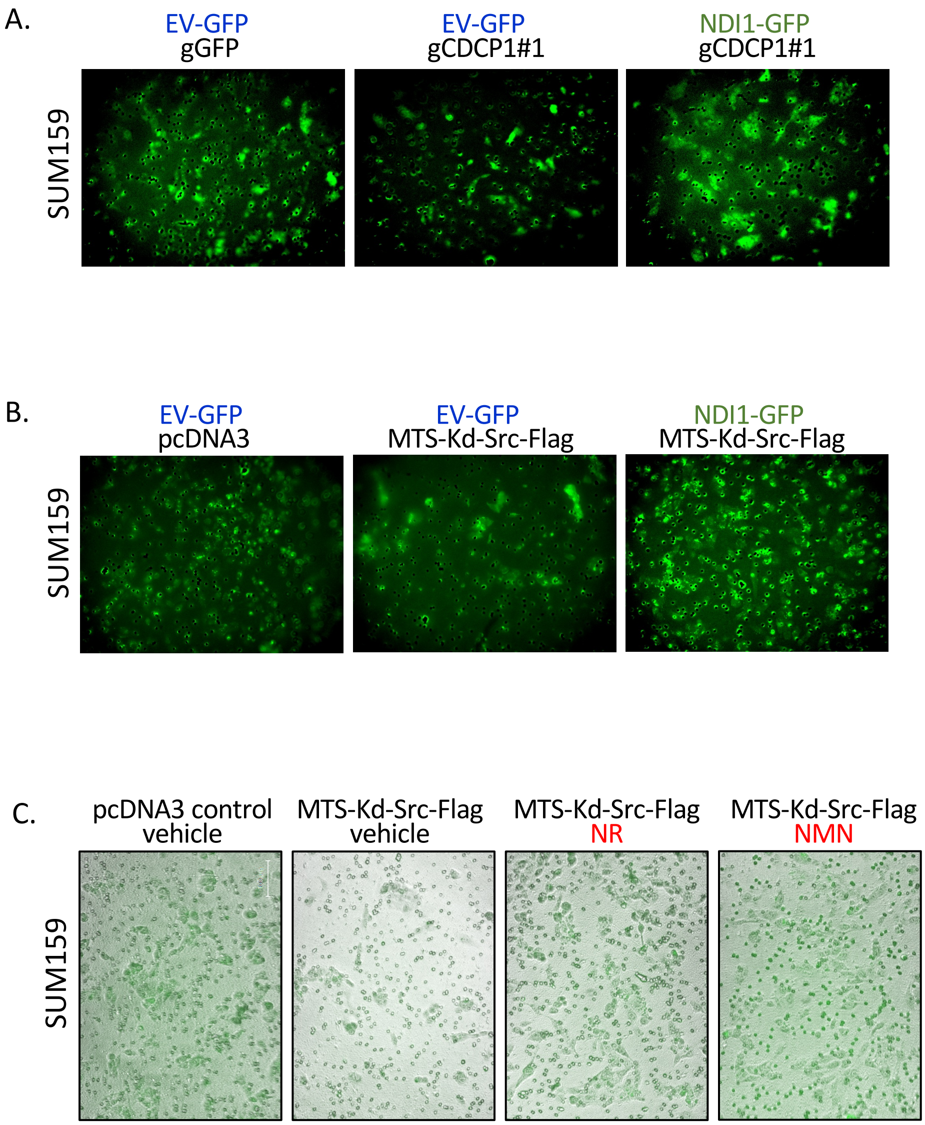

Fig. 11 | CDCP1/mitochondrial Src axis stimulates TNBC migration through NAD^+-generating function of Complex I. Representative transwell images associated with **Figure 6**. **A)** NDI1 overexpression partially rescues migration in CDCP1 knockout SUM159 cells (n=3). **B)** NDI1 overexpression rescues migration in MTS-Kd-Src-Flag SUM159 cells (n=3). **C)** NAD^+ boosters Nicotinamide Riboside (NR) and Nicotinamide mononucleotide (NMN) rescue migration in MTS-Kd-Src-Flag EV-GFP SUM159 cells (n=3, 0.5 mM NR or NMN treated overnight). All transwell images were taken at 20x.

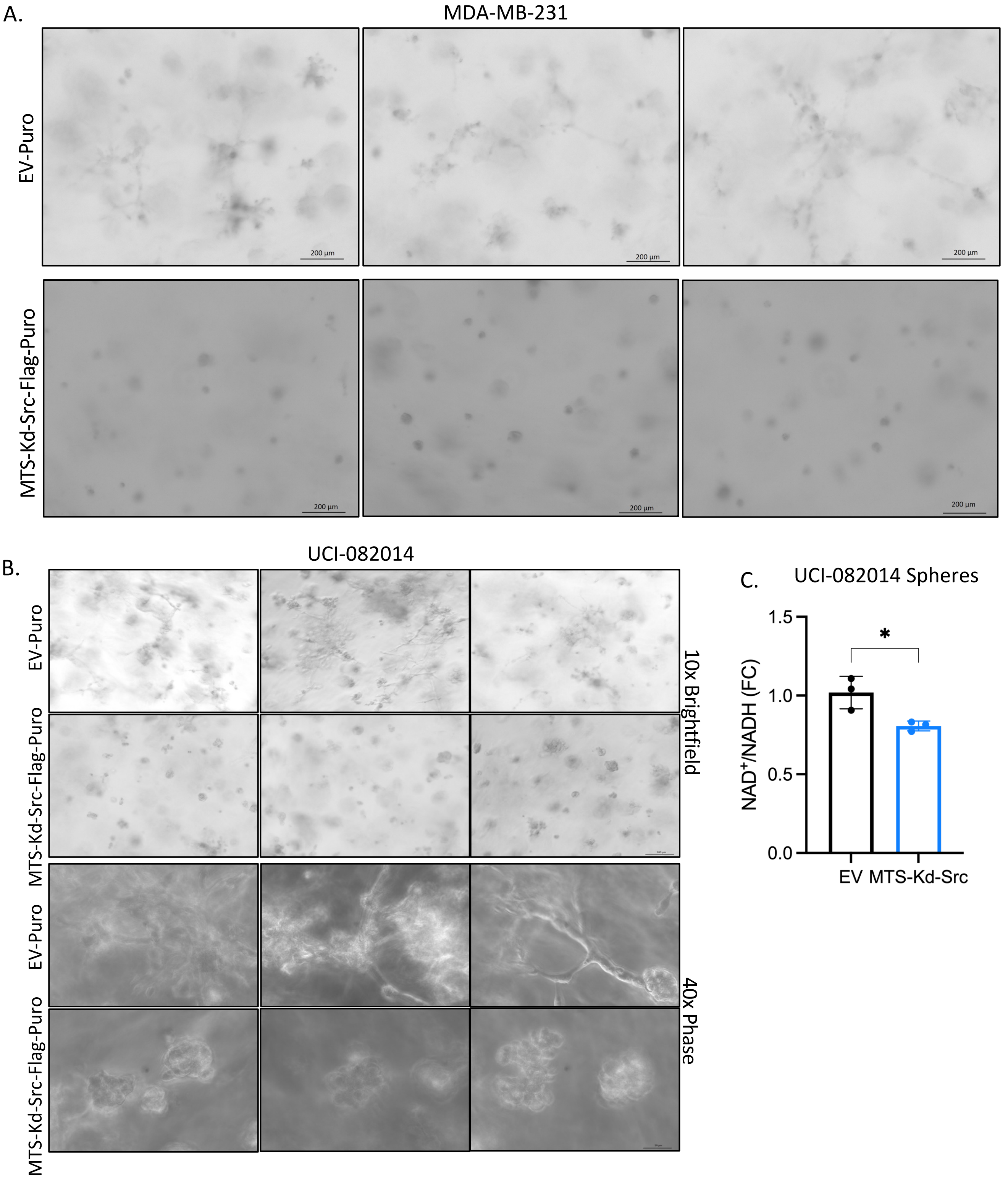

Supplemental Fig. 12 | MTS-Kd-Src decreases invasion in TNBC cells. A) Representative brightfield images of MDA-MB-231 spheres expressing either EV-Puro or MTS-Kd-Src-Puro. **B)** Representative brightfield and phase images of UCI-082014 spheres expressing either EV-Puro or MTS-Kd-Src-Puro. For 10x brightfield magnification, scale bar = 200 microns, 40x magnification, scale bar = 50 microns. C. NAD^+/NADH ratio is significantly reduced in UCI-082014 spheres expressing MTS-Kd-Src-Flag-Puro compared to EV-Puro spheres (n=3). P values were analyzed by student's t-test, error bars represent SEM; *P<0.05.

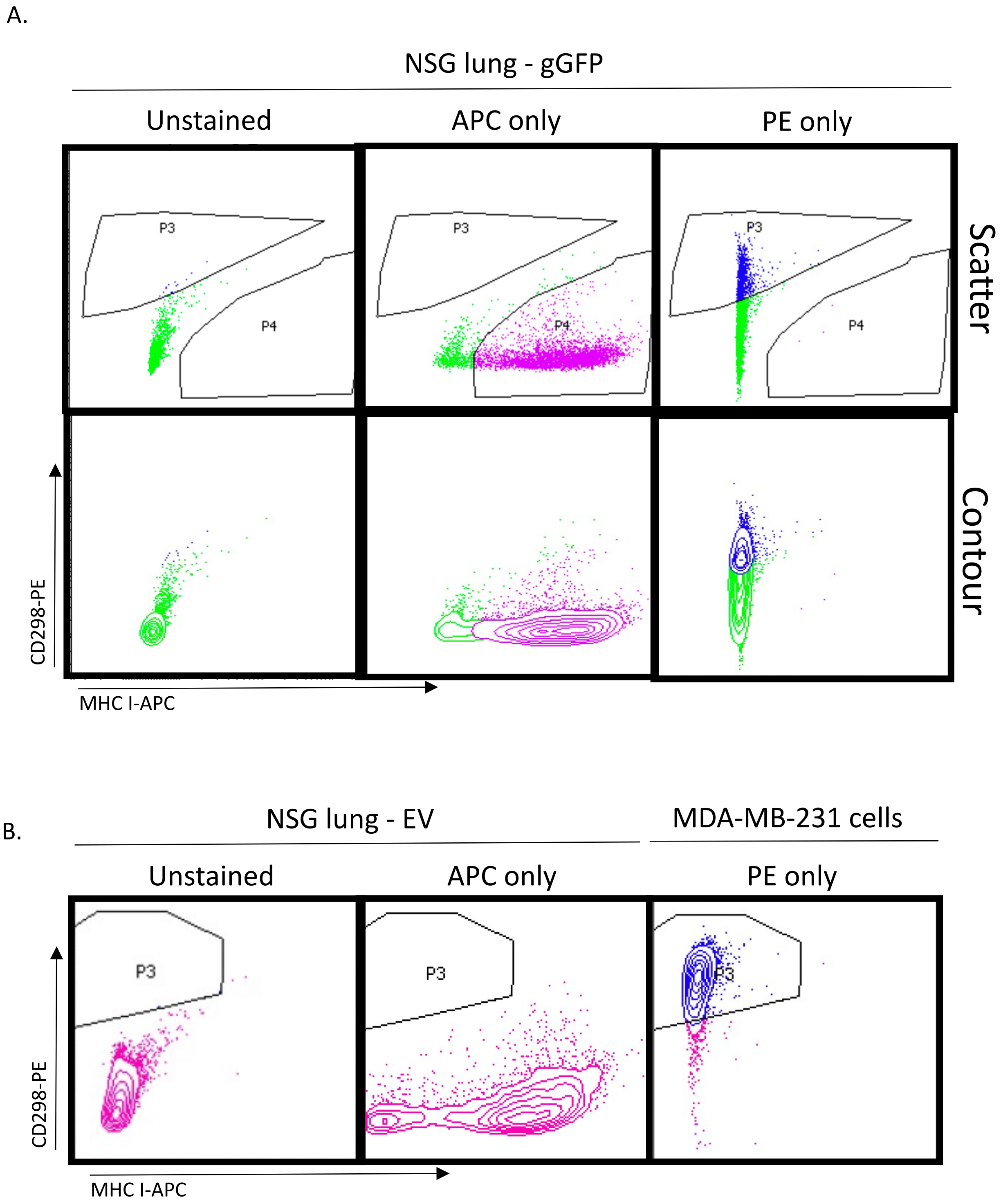

Supplemental Fig. 13 | Compensation controls for mouse metastasis studies. Gating strategies for unstained, APC and PE single stains for **A)** CDCP1 knockout metastasis study (using gGFP for compensation controls) and **B)** mitochondrial-localized kinase-dead Src metastasis study (using EV as compensation controls). Note in **(B)** that pure MDA-MB-231 cells from cell culture were used as PE control to accurately visualize PE+ population. Otherwise, mouse lung tissue was used for remaining compensation controls.
